# Interplay of the nasal microbiome and epigenome among adolescents

**DOI:** 10.1186/s13148-026-02093-1

**Published:** 2026-02-27

**Authors:** Anne K. Bozack, Javier Perez-Garcia, Sheryl Rifas-Shiman, Yanjiao Zhou, Joanne Sordillo, Jenny Jyoung Lee, Brent Coull, Peggy S. Lai, Emily Oken, Marie-France Hivert, Diane R. Gold, Andres Cardenas

**Affiliations:** 1 Division of Epidemiology, Department of Population Health, NYU Grossman School of Medicine, New York, NY USA; 2 Department of Epidemiology and Population Health, Stanford University School of Medicine, Stanford, CA USA; 3 Division of Chronic Disease Research Across the Lifecourse, Department of Population Medicine, Harvard Medical School, Harvard Pilgrim Health Care Institute, Boston, MA USA; 4 Department of Medicine, UConn Health, Farmington, CT USA; 5 VA Boston Healthcare Center, Boston, MA USA; 6 Division of Artificial Intelligence and Data Science, Ewha Womans University, Seoul, Republic of Korea; 7 Department of Biostatistics, Harvard T.H. Chan School of Public Health, Boston, MA USA; 8 Division of Pulmonary and Critical Care Medicine, Massachusetts General Hospital, Boston, MA USA; 9 Diabetes Unit, Massachusetts General Hospital, Boston, MA USA; 10 Department of Environmental Health, Harvard T. H. Chan School of Public Health, Boston, MA USA; 11 Department of Medicine, Channing Division of Network Medicine, Brigham and Women’s Hospital, Boston, MA USA; 12 Department of Medicine, Harvard Medical School, Boston, MA USA; 13 Department of Pediatrics, Stanford University School of Medicine, Stanford, CA USA

**Keywords:** Respiratory microbiome, Nasal microbiome, Nasal DNA methylation, Epigenetic aging, Multi-omics

## Abstract

**Background:**

The respiratory microbiome, including that of the nasal cavity, is involved in host defense and airway pathophysiology. Interactions of the microbiome with the host immune system may impact health and disease susceptibility through changes in the epigenome. In this study, we aimed to analyze cross-sectional associations of nasal microbiome composition and the nasal epigenome among adolescents in the Project Viva cohort (*N* = 372, mean age: 13.0 years). We collected nasal swabs from anterior nares, profiled the microbiome by 16 S rRNA gene sequencing, and grouped samples into 6 clusters using partitioning around medoids. Nasal cell DNA methylation was measured with the Illumina MethylationEPIC BeadChip. In an epigenome-wide association study, we tested for associations of microbiome cluster assignment and DNA methylation using robust linear models adjusting sociodemographics, season, batch, and surrogates of cell type composition. Among significant loci, we conducted differential abundance analysis to identify individual bacterial genera associated with DNA methylation levels.

**Results:**

A total of 45 loci had differential methylation between two or more microbiome clusters (*p*_*Bonferroni*_< 0.05). Methylation differences between clusters ranged from 0.20 to 12.45% (median = 0.95%). Differentially methylated loci were near genes related to asthma (*ITPR2*, *MAPK1*), lung function (*FKBP11*), mitochondrial function (*MRPL20*, *SPTBN1*), inflammation (*C3*), and immune function (*N4BP3*, *EIF5*). The abundance of individual taxa, particularly *Propionibacterium*, was associated with methylation at 15 of these loci (*FDR* < 0.05). In addition, we found greater *Corynebacterium* abundance was associated with lower nasal epigenetic aging (*FDR* < 0.05).

**Conclusions:**

Our findings support the hypothesis that the nasal microbiome is associated with small-to-modest variation in the nasal epigenome. Future research is needed to investigate how the relationship between the nasal microbiome and epigenome is impacted by environmental exposures, as well as the health effects of microbial and epigenetic variation in early life and across the life course.

**Supplementary Information:**

The online version contains supplementary material available at 10.1186/s13148-026-02093-1.

## Background

Respiratory illnesses, including bronchitis, pneumonia, and asthma, are among the leading causes of childhood hospitalizations [1], and respiratory infections early in life can have adverse effects on lung function later in life [2]. Changes in the microbiota and epigenome of the respiratory tract may be involved in linking exogenous and endogenous factors to health [3]. The human microbiome plays an important role in health and disease through interactions with the host immune system [4], and dysbiosis of the respiratory microbiome may be involved in disease etiology [5]. Host-microbiome interactions may impact respiratory function and disease susceptibility through epigenetic programming, having implications for long-term health [6].

The microbiome of the lower airways and lungs is dependent on the migration of bacteria from the upper respiratory tract and host microbial defenses [7]. In ways that are poorly understood, the microbial composition of both upper and lower airways is likely related to airway epithelial barrier responses to environmental or pathogenic exposures [8, 9]. In tandem with the development of the immune system, establishment of the respiratory microbiome begins immediately after birth [7], and the respiratory microbiome matures throughout childhood to that characteristic of adult microbial communities [10]. The composition of the early life respiratory tract microbiome is dynamic and influenced by the external and host environment [10]. Epithelial cells and mucosal surfaces, which line the respiratory tract and act as an interface between exogenous microbes and the host, also impact microbiome composition through limiting microbial migration and through innate and adaptive immune response [7, 11].

Animal and human studies have demonstrated interactions between respiratory microbiome colonization, inflammatory markers, and respiratory diseases [4]. Bacterial diversity and composition of the upper and lower respiratory tracts may influence health and disease [10, 12]. Decreased alpha diversity of the upper airways has been associated with decreased lung function and increases in pathogenic species [12, 13]. Imbalances of microbiota may induce inflammation, contributing to the development or exacerbation of respiratory diseases such as asthma [14, 15]. The nasal epigenome has also been associated with respiratory health in children [16]. In the current study population of adolescents, both the nasal microbiome and nasal epigenome have been associated with environmental exposures and respiratory health [17, 18]. Microbiome profiles (based on clustering) and genus-level abundance were associated with respiratory outcomes, including aeroallergen sensitization, inflammation biomarkers, and lung function [18]. DNA methylation (DNAm) at individual loci and regions at genes involved in immune response was associated with asthma and biomarkers of allergic disease [17]. Microbiome-immune interactions may be mediated in part by epigenetic regulation, including changes in DNAm [6, 19]. However, there is a scarcity of research investigating the interplay between the respiratory microbiome and epigenome.

**Fig. 1:**
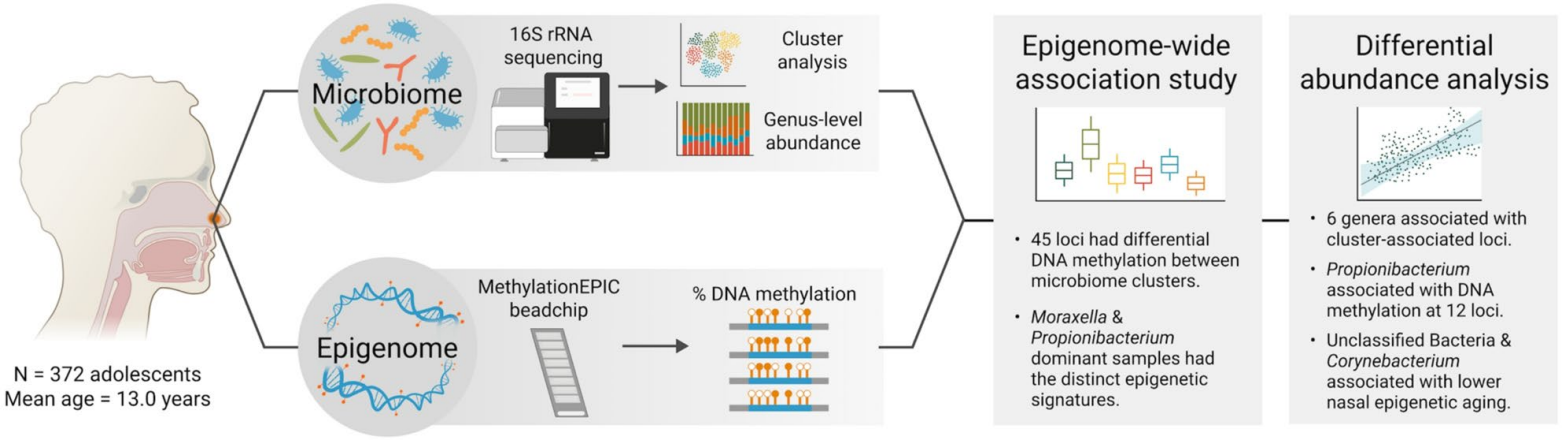
Study overview

To address this gap, we aimed to test associations of nasal microbiome composition and the nasal epigenome measured in the anterior nares of adolescents enrolled in the Project Viva prebirth cohort (summarized in Fig. 1). In a cross-sectional analysis using a hypothesis-free approach, we tested for epigenome-wide associations of DNAm levels and microbiome clusters characteristic of common nasal microbial profiles. To determine if specific taxa were driving associations with microbiome clusters, we investigated associations of DNAm and the abundance of individual bacterial genera. In addition, we analyzed the extent to which microbiome clusters and microbial abundance were associated with epigenetic age deviation, a biomarker of biological development and aging sensitive to immune cell variation.

## Methods

### Study population

This study was based in Project Viva, a prospective pre-birth cohort study developed to understand the effects of maternal diet and other factors on pregnancy and child health [20–22]. The current study used cross-sectional data. Project Viva recruited mothers between 1999 and 2002 during their first prenatal visit at Atrius Harvard Vanguard Medical Associates, a group practice in Massachusetts, US. Mothers were eligible if they were fluent in English, < 22 weeks gestation at the first prenatal visit, and had a singleton pregnancy. The initial cohort included 2128 live births. The Project Viva research team re-contacted mothers and children at periodic postnatal follow-up visits including an Early Teen visit (mean age: 12.9 years). Mothers provided informed consent at recruitment and postpartum visits. Beginning at the mid-childhood visit, verbal assent was also obtained from the child. All study protocols were approved by the Institutional Review Board of Harvard Pilgrim Health Care (IRB reference # 235301) in line with ethical standards established by the Declaration of Helsinki. The current study followed the Strengthening the Organization and Reporting of Microbiome Studies (STORMS) reporting guidelines (Supplemental Table S1) [23].

Trained research assistants collected maternal sociodemographics through interviews and self-administered questionnaires, as well as child height and weight. We calculated age- and sex-specific body mass index (BMI) *z*-scores based on US National Reference data. At the Early Teen visit, mothers reported if their child had ever been diagnosed with asthma, if their child had wheezing in the past 12 months, and if their child had used medication for breathing problems, including beta-agonists, corticosteroids, and nasal steroids, in the past 12 months. If mothers reported “yes” to asthma diagnosis and wheezing or medication use in the past 12 months, the child was classified as having current asthma; if mother reported “yes” to asthma diagnosis but no wheezing or medication use in the past 12 months, the child was classified as having former asthma. On the age 19 questionnaire, teenagers self-reported their race and ethnicity.

### Sample collection

Swabs of anterior nares have been shown to be a noninvasive sample method yielding nasal epithelial cells [24]. At the early-teen visit, trained technicians collected nasal swabs from the anterior nares of adolescents (*N* = 547) using sterile cotton swabs (Supplemental Fig. S1). Technicians were instructed not to collect nasal samples on children who reported “feeling sick today” or who reported symptoms that technicians judged were only due to allergies. Immediately after sample collection, swabs were placed in DNA lysis buffer (Promega, Madison, WI, US) and frozen. DNA was isolated with the Maxwell 16 Buccal Swab LEV DNA Purification Kit (Promega, Madison, WI, US) according to the manufacturer’s instructions. Samples were stored at − 80° until analysis.

### Nasal microbiome sequencing

Methods for measurement and quality control of nasal microbiome and epigenome data included in the *Supplemental Methods* and summarized below.

A subset of 436 nasal swab samples was selected for microbiome sequencing, which was conducted at the Jackson Laboratory (Farmington, CT). The V1-V3 regions of bacterial 16S ribosomal RNA (rRNA) gene were amplified from genomic DNA using 27F (5′-AGAGTTTGATCCTGGCTCAG-3′) and 534R primers (5′-ATTACCGCGGCTGCTGG-3′). 16S libraries were sequenced using an Illumina MiSeq and 2 × 300 v3 sequencing kit (Illumina, San Diego, CA) with dual distinct barcodes. Negative and positive extraction and library controls were included.

Data processing and quality control (QC) included demultiplexing; removal of sequences with low quality, ambiguous bases, and 16 S primers; assembly of paired-end sequences; and removal of chimeric sequences. 16S amplicons were clustered into operational taxonomic units (OTUs) at 97% sequence identity levels, and taxonomic classification for each OTU. We performed QC of OTUs including decontamination at a probability threshold of 0.1, excluding samples with low read counts (< 10,000 reads), and removing low-abundance OTUs (< 10 copies in individual samples).

### Nasal DNA methylation measurement

Measurement of nasal DNAm has previously been described [17]. DNA underwent bisulfite conversion and DNAm was measured using the Infinium MethylationEPICv1 BeadChip (Illumina, San Diego, CA, US). We performed QC of DNAm data including dropping samples with low intensities, samples with a mismatch between recorded and predicted sex, and samples that failed a genotype check.

Probe-level QC included filtering based on detection *p*-values, probes annotated to sex chromosomes, non-CpG probes, cross-reactive probes, and probes with a SNP within two base pairs of the target site or at the single base pair extension. We also removed probes exhibiting a trimodal distribution of methylation values characteristic of a SNP. Data were normalized using functional normalization and batch effects due to plate were adjusted for using *ComBat* [25]. We calculated Reference-Free Adjustment for Cell-Type composition (ReFACTor) [26] components to control for cellular heterogeneity.

### Statistical analysis

*Microbiome cluster analysis*: Before conducting cluster analysis of microbiome samples, we performed rarefaction to control for uneven sequencing depth between samples. To minimize artificial uncertainty that may be created through rarefying data [27], rarefaction was performed with 1,000 iterations of resampling the data at the minimum library size and averaging the OTU read counts across iterations. OTUs assigned to the same taxa at the genus level were merged, resulting in 322 OTUs for analysis. We performed clustering of microbiome samples to identify clusters characteristic of common nasal microbial compositions. First, we evaluated several beta diversity measures, considering both Manhattan-based measures, which prioritize variation in high-abundance OTUs, and Kullback-Leibler (KL)-based measures, which prioritize low-abundance OTUs [28], including Jensen-Shannon divergence (JSD), Bray-Curtis divergence, Jaccard distance, and Jensen-Shannon distance (i.e., the square root of JSD). For each measure, we plotted the silhouette index and Calinski-Harabasz index (CHI) calculated using 2–10 clusters. We chose to use the KL-based JSD using six clusters to maximize the clustering metrics. Clustering was performed using partitioning around medoids (PAM), which is more robust to outliers than k-means clustering. Separation of clusters was visually inspected using ordination plots (Supplemental Fig. S2). We also assessed the stability of clusters to outliers using leave-one-out for individual samples. For each iteration, we conducted PAM using the JSD and selected the number of clusters based on the maximum silhouette index. The leave-one-out sample was assigned to a cluster based on the medoid with the minimum distance. Clusters were named based on the number of samples, with Cluster 1 being the largest cluster. Cluster assignments were compared by calculating the Adjusted Rand Index (ARI) for PAM using all samples versus each leave-one-out iteration. For all iterations, six clusters were selected, and the ARI = 0.99, indicating high agreement and minimal impact of individual samples. Cluster assignments across iterations were also assessed visually (Supplemental Fig. S3).

Nasal microbiome and epigenome data were available for a total of 375 samples (Supplemental Fig. S1). We excluded three samples due to missing covariate data, and therefore analyses used 372 samples. Prior to rarefaction, we calculated alpha diversity measures. We calculated descriptive statistics (frequency and percent for categorical variables and mean and standard deviation (SD) for continuous variables) for adolescents included in the study. Differences in characteristics between microbiome clusters were evaluated using Fisher’s exact test and Kruskal-Wallis rank sum test.

*Epigenome-wide association study (EWAS)*: We modeled DNAm levels as M-values (logit_2_ transformation of Beta-values) to better meet model assumptions [29]. We tested for associations of microbiome cluster assignment and DNAm using robust linear models implemented in *limma* [30]. We adjusted models for sine and cosine of season to control for annual and biannual trends, sex, age at sample collection, race and ethnicity, BMI *z*-score, maternal education (college graduate or greater vs. not a college graduate), 10 ReFACTor components, and 16S batch-run. To conduct pairwise contrasts between all clusters, we constructed a contrast matrix and estimated coefficients and standard errors using the *contrasts.fit* function. We used the *decideTests* function with the “global” option to calculate all pairwise contrasts. A Bonferroni correction was used to control for family-wise error rate with a significance threshold of *p*_*Bonferroni*_ < 0.05, which is the equivalent to *p* < 4.66 × 10^−9^, i.e., 0.05/(715,023 CpGs × 15 pairwise contrasts). To evaluate inflation of test statistics, for each cluster contrast, we calculated the genomic inflation factor (λ) and the Bayesian inflation factor (BIF), which is based on an estimation of the empirical null distribution of *p*-values to avoid overestimation of inflation [31]. For interpretation of results, effect sizes in Δ M-values were converted to Δ Beta-values using the M-model-M-mean method of Xie et al. [32]. We used the Illumina MethylationEPICv1 BeadChip manifest to identify genes mapped to cluster-associated CpGs.

We conducted Gene Ontology (GO) [33, 34] enrichment analysis using *gometh* to adjust for bias introduced due to differential representation of genes on the MethylationEPIC array [35, 36]. For each microbiome cluster contrast, CpGs associated with microbiome cluster assignment at a false discovery rate (FDR) adjusted *p*-value < 0.05 were used as the input to test for gene ontology enrichment. GO terms including more than 1 differentially methylated gene and with *p* < 0.05 were considered having suggestive associations with microbiome clusters.

To evaluate if differentially methylated positions (DMPs) were influenced by genetic variants, i.e., methylation quantitative trait loci (meQLTs), we looked up DMPs in the EPIGEN MeQTL Database [37]. The EPIGEN MeQTL Database reports cis and trans meQTLs that were identified in an analysis of three cohorts with DNAm in blood measured using the MethylationEPIC array. We also visually inspected the distribution of methylation values for each DMP using density and scatter plots. To test if meQTLs may be differentially distributed across microbiome clusters, we grouped methylation Beta-values using K-means and tested for independence of methylation groups and microbiome clusters using Chi-squared tests.

We conducted sensitivity analyses to test for the influence of asthma medication use and outliers in DNAm data. First, we repeated the EWAS of associations of microbiome cluster assignment and DNAm adjusting for use of medication for breathing problems in the past 12 month. Second, for each CpG site, we winsorized M-values by replacing values < the 5th or > the 95th percentile with the next closest value. We then repeated the EWAS using the winsorized DNAm data. In addition, to evaluate if our cluster-associated signals were unlikely to be due to random variation, we estimated the empirical null distribution of DMPs significant in at least one pairwise contrast (*p*_*Bonferroni*_ < 0.05) by performing 500 EWAS permutations with random cluster assignments. As in our primary analysis, EWAS were performed using fully adjusted robust linear models implemented in *limma.* The empirical *p*-value from permutation testing was calculated as (1 + sum(number of DMPs from random cluster assignment > number of observed DMPs))/(500 + 1).

*Differential abundance analysis*: To test if the abundance of individual bacterial genera were driving associations of microbiome clusters with DNAm, we conducted differential abundance analysis. We applied Analysis of Compositions of Microbiomes with Bias Correction 2 (ANCOM-BC2), which controls for sample-specific and taxon-specific biases and regularizes variance [38]. Because ANCOM-BC2 log-transforms observed count data, a pseudo-count is added prior to transformation, and sensitivity analyses are conducted with an array of pseudo-counts. ANCOM-BC2 was run on microbiome data prior to rarefaction, with taxa collapsed at the genus level and filtering for taxa present ≥ 15% of samples (39 taxa). DNAm levels of each DMP identified in the EWAS were modeled as the independent variables as M-values. We adjusted analyses for sex, age at sample collection, race and ethnicity, BMI *z*-score, sine and cosine of season, maternal education, 10 ReFACTor components, and 16S batch-run. FDR correction was performed for each DMP (39 tests). Taxa associated with DNAm at *FDR* < 0.05 and that passed sensitivity analysis for pseudo-counts were considered statistically significant. Effect sizes were expressed as log(fold change) (logFC) per IQR increase in M-value.

*Associations with epigenetic aging*: We evaluated associations of nasal microbiome clusters and epigenetic age deviation (EAD), also known as epigenetic age acceleration. We calculated Horvath pan-tissue epigenetic age [39] from nasal microbiome DNAm using Horvath’s new online calculator (https://dnamage.g.enetics.ucla.edu/newonlinecalculator) with normalization. The Horvath pan-tissue clock was selected because its training data encompassed a broad range of human tissues and included samples collected from children and adolescents. EAD is calculated by the website as the residuals of regression epigenetic age on chronological age. Associations of microbiome cluster assignment and EAD were tested using linear models adjusting for sex, age at sample collection, race and ethnicity, BMI *z*-score, sine and cosine of season, maternal education, and 16S batch-run. Pairwise contrasts were conducted using the *emmeans* R package. Associations of EAD and the abundance of individual bacterial genera were also tested using ANCOM-BC2 adjusting for the same set of covariates, and associations with *FDR* < 0.05 and that passed sensitivity analysis were considered statistically significant.

All analyses were conducted in R version 4.4.1 [40].

## Results

### Participant characteristics and nasal microbiome clusters

A subset of 372 adolescents enrolled in Project Viva with complete nasal microbiome, epigenome, and covariate data collected at the early-teen follow-up visit were included in this study (Supplemental Fig. S1). Approximately half of participants were female (51.1%) and 66.9% were non-Hispanic White (Table 1). Participants had a mean (SD) age of 13.0 (0.7) years and a BMI z-score of 0.44 (1.10). A total of 98 (26.3%) and 39 (10.5%) of participants reported having current asthma or using asthma medication in the past 12 months, respectively. At enrollment in Project Viva, most mothers had completed college or higher education (69.4%), with a majority having a household income of $70,000 or greater (59.9%).

**Table 1:** Characteristics of children included in the study by nasal microbiome cluster. Values reported as n (%) for categorical variables or mean (standard deviation) for continuous variables

	All participants	Cluster 1 corynebacterium dominant	Cluster 2 propionibacterium dominant	Cluster 3 staphylococcus dominant	Cluster 4 staphylococcus and streptococcus dominant	Cluster 5 unclassified neisseriaceae dominant	Cluster 6 moraxella dominant	*p* ^a^
*N*	372	120	83	79	41	33	16	
Female, n (%)	190 (51.1%)	61 (50.8%)	46 (55.4%)	35 (44.3%)	27 (65.9%)	13 (39.4%)	8 (50.0%)	0.28
Age, mean (SD)	13.0 (0.7)	12.9 (0.6)	13.3 (0.8)	12.8 (0.5)	13.0 (0.69)	13.0 (0.8)	13.1 (0.8)	< 0.001
BMI z-score, mean (SD)	0.44 (1.10)	0.50 (1.07)	0.76 (0.90)	0.44 (1.16)	− 0.01 (1.15)	0.18 (1.10)	0.07 (1.36)	0.003
Race and ethnicity, n (%)								0.5
Asian	7 (1.9%)	1 (0.8%)	3 (3.6%)	2 (2.5%)	1 (2.4%)	0 (0%)	0 (0%)	
Black	57 (15.3%)	20 (16.7%)	14 (16.9%)	8 (10.1%)	4 (9.8%)	7 (21.2%)	4 (25.0%)	
Hispanic	35 (9.4%)	11 (9.2%)	12 (14.5%)	7 (8.9%)	3 (7.3%)	2 (6.1%)	0 (0%)	
White	249 (66.9%)	79 (65.8%)	44 (53.0%)	60 (75.9%)	32 (78.0%)	23 (69.7%)	11 (68.8%)	
> 1 race or other	24 (6.5%)	9 (7.5%)	10 (12.0%)	2 (2.5%)	1 (2.4%)	1 (3.0%)	1 (6.3%)	
Asthma, n (%)								0.53
Current	47 (12.6%)	16 (13.3%)	11 (13.3%)	8 (10.1%)	4 (9.8%)	7 (21.2%)	1 (6.3%)	
Former	51 (13.7%)	17 (14.2%)	10 (12.0%)	13 (16.5%)	4 (9.8%)	6 (18.2%)	1 (6.3%)	
Missing	4 (1.1%)	1 (0.8%)	1 (1.2%)	2 (2.5%)	0 (0%)	0 (0%)	0 (0%)	
Asthma medication use in past 12 months, n (%)	39 (10.5%)	14 (11.7%)	8 (9.6%)	6 (7.6%)	3 (7.3%)	7 (21.2%)	1 (6.3%)	0.52
Missing	4 (1.1%)	3 (2.5%)	1 (1.2%)	0 (0%)	0 (0%)	0 (0%)	0 (0%)	
Maternal age at enrollment, mean (SD)	32.1 (5.3)	32.4 (5.2)	31.2 (5.3)	33.0 (5.3)	31.9 (5.7)	31.8 (4.6)	30.2 (6.5)	0.35
Mother college graduate or higher, n (%)	258 (69.4%)	85 (70.8%)	57 (68.7%)	58 (73.4%)	31 (75.6%)	22 (66.7%)	5 (31.3%)	0.06
Household income > $70,000/year, n (%)	223 (59.9%)	72 (60.0%)	45 (54.2%)	54 (68.4%)	25 (61.0%)	20 (60.6%)	7 (43.8%)	0.36
Missing	34 (9.1%)	4 (11.7%)	4 (4.8%)	4 (5.1%)	4 (9.8%)	6 (18.2%)	2 (12.5%)	
Richness, mean (SD)	45.8 (41.0)	37.2 (31.0)	27.3 (24.0)	45.1 (36.2)	109.0 (46.8)	52.2 (39.9)	33.8 (31.0)	< 0.001
Shannon index, mean (SD)	1.62 (0.82)	1.41 (0.49)	1.37 (0.55)	1.39 (0.76)	3.15 (0.71)	1.79 (0.66)	1.26 (0.48)	< 0.001
Simpson diversity index, mean (SD)	0.63 (0.20)	0.60 (0.15)	0.59 (0.18)	0.54 (0.24)	0.89 (0.13)	0.71 (0.13)	0.54 (0.20)	< 0.001

^a^*P*-values calculated using Fisher’s exact test for categorical variables or Kruskal-Wallis rank sum test for continuous variables

**Fig. 2:**
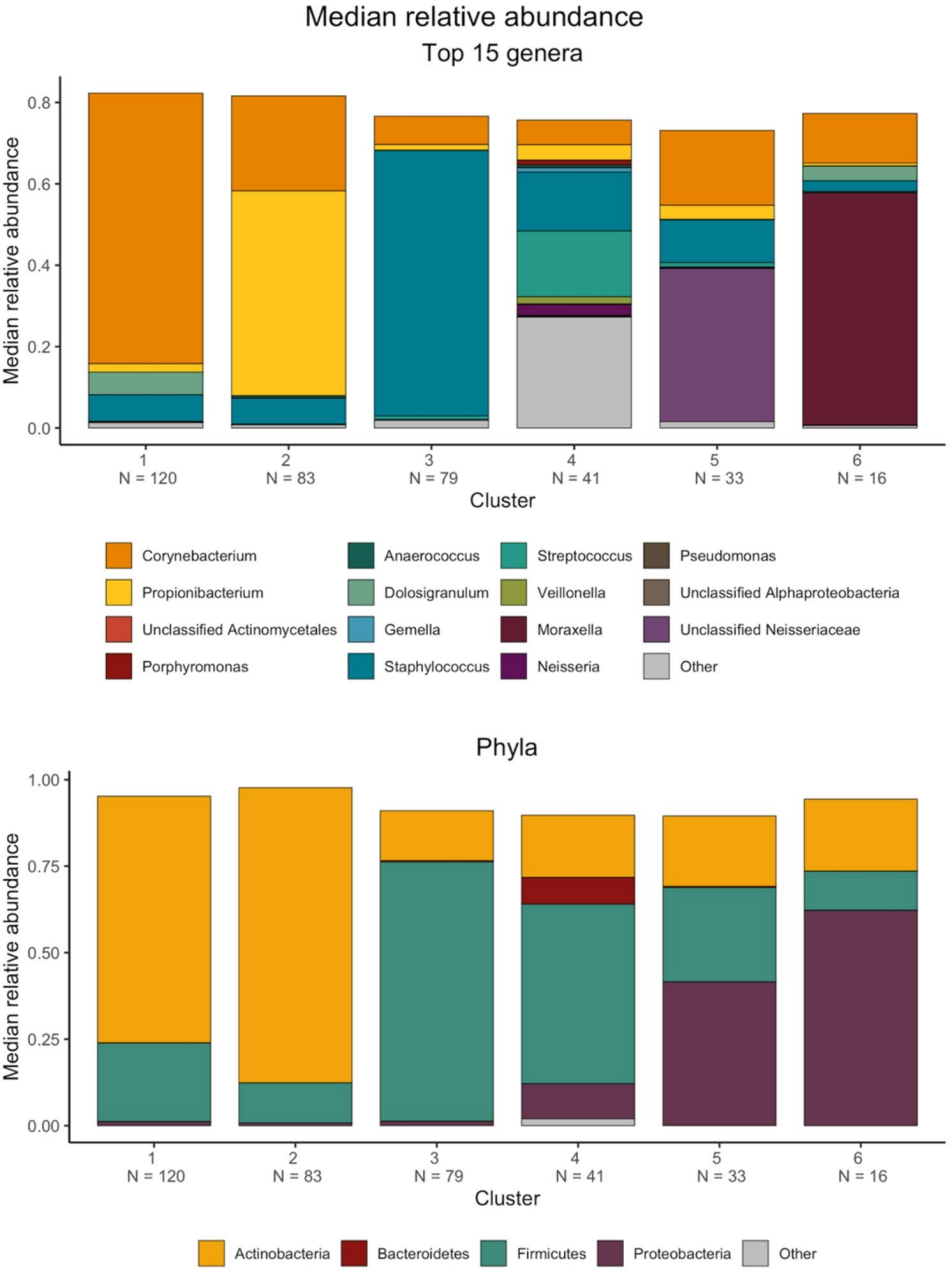
Nasal microbiome cluster abundance by genera and phyla. Partitioning around medoids (PAM) was used to cluster samples based on the Jensen-Shannon distance (JSD). The six resulting clusters represented distinct clusters: Cluster 1: *Corynebacterium* dominant; Cluster 2: *Propionibacterium* dominant; Cluster 3: *Staphylococcus* dominant; Cluster 4: *Staphylococcus* and *Streptococcus* dominant; Cluster 5: unclassified *Neisseriaceae* dominant; and Cluster 6: *Moraxella* dominant

We profiled the nasal microbiome by targeted sequencing of the V1-3 region of the 16S rRNA gene and grouped amplicons into OTUs. Samples were grouped into six clusters using PAM based on the JSD (Supplemental Fig. S2). The six microbiome clusters had distinct microbial compositions as demonstrated by the median relative abundance (RA) of the top genera (Fig. 2): Cluster 1 (*N* = 120 participants): *Corynebacterium* dominant (median RA = 67%); Cluster 2 (*N* = 83 participants): *Propionibacterium* dominant (median RA = 50%); Cluster 3 (*N* = 79 participants): *Staphylococcus* dominant (median RA = 65%); Cluster 4 (*N* = 41 participants): *Staphylococcus* and *Streptococcus* dominant (median RA = 14% and 16%, respectively); Cluster 5 (*N* = 33 participants): unclassified *Neisseriaceae* dominant (median RA = 38%); and Cluster 6 (*N* = 16 participants): *Moraxella* dominant (median RA = 57%). In Cluster 4, less abundant genera (i.e., not represented by the top 15 genera) had an overall median RA of 27%. The dominant phyla were *Actinobacteria* in Clusters 1 and 2 (median RA = 71% and 85%, respectively), *Firmicutes* in Clusters 3 and 4 (median RA = 75% and 52%, respectively), *Proteobacteria* and *Firmicutes* in Cluster 5 (median RA = 41% and 27%, respectively), and *Proteobacteria* in Cluster 6 (median RA = 62%). Adolescents in Cluster 2 had the greatest mean age and BMI *z*-score (Table 1); participant characteristics otherwise did not differ between clusters. Cluster 4 had the greatest mean richness (109) and alpha diversity as represented by the Shannon index (3.15) and Simpson diversity index (0.89), whereas Cluster 6 had the lowest mean richness (33.8) and Shannon index (1.26). Clusters 3 and 6 had the lowest mean Simpson diversity index (0.54).

## Microbiome clusters are associated with nasal DNA methylation

We tested for associations of microbiome cluster assignment and DNAm using adjusted robust linear models with pairwise contrasts. Q-Q plots, genomic inflation values (λ), volcano plots, and Manhattan plots for each contrast are shown in Supplemental Fig. S4. We observed some evidence of genomic inflation, particularly for contrasts including the smallest cluster, Cluster 6 (*Moraxella* dominant) (λ = 1.37–1.67); however, the Bayesian inflation factor (BIF), which is more valid in the presence of many small effects [31], showed less inflation (BIF = 1.10–1.14).

**Fig. 3:**
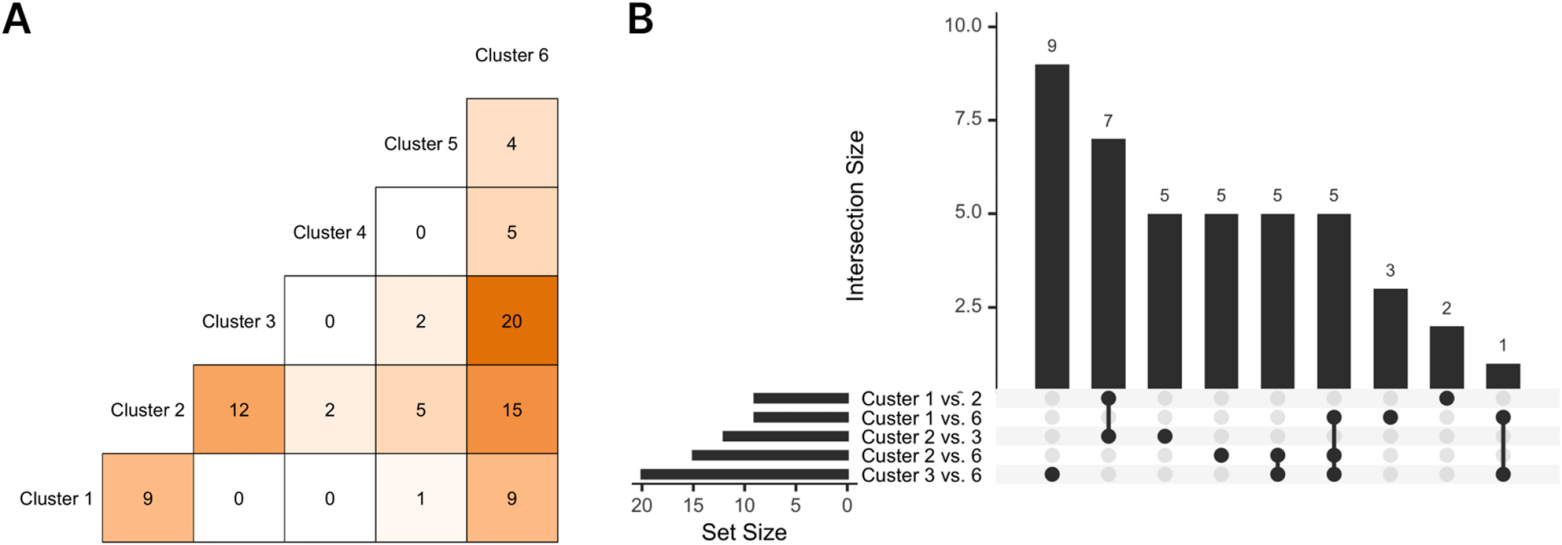
Summary of result from epigenome-wide association study of microbiome clusters and DNA methylation.** A** Number of differentially methylated positions (DMPs) associated with each microbiome cluster contrast (*p*_*Bonferroni*_ < 0.05). **B** UpSet plot of common DMPs associated with multiple cluster contrasts. Results from robust linear models adjusted for sine and cosine of season to control for annual and biannual trends, age at sample collection, race and ethnicity, BMI *z*-score, maternal education, 10 ReFACTor components, and 16S batch-run. Cluster 1: *Corynebacterium* dominant; Cluster 2: *Propionibacterium* dominant; Cluster 3: *Staphylococcus* dominant; Cluster 4: *Staphylococcus* and *Streptococcus* dominant; Cluster 5: unclassified *Neisseriaceae* dominant; and Cluster 6: *Moraxella* dominant

**Fig. 4:**
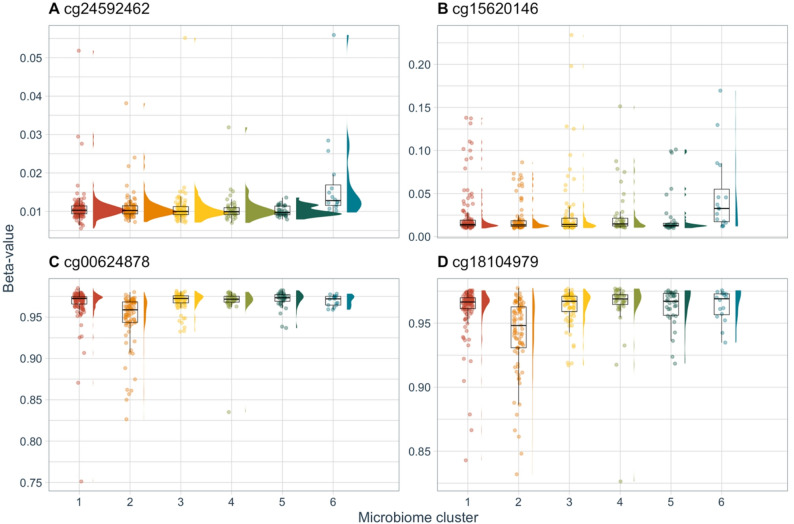
Beta-values of selected differentially methylated positions (DMPs) (*p*_*Bonferroni*_ < 0.05).** A** cg24592462 (*FOXJ3*): hypermethylated in Cluster 6 versus Clusters 1, 2, 3, 4, and 5. **B** cg15620146 (*FKBP11*): hypermethylated in Cluster 6 versus Clusters 1, 2, 3, 4, and 5. **C** cg00624878 (*CREBBP*): hypomethylated in Cluster 2 versus Clusters 1, 3, 4, and 5. **D** cg18104979 (*INPP5D*): hypomethylated in Cluster 2 versus Clusters 1, 3, 4, and 5. Results from robust linear models adjusted for sine and cosine of season to control for annual and biannual trends, age at sample collection, race and ethnicity, BMI *z*-score, maternal education, 10 ReFACTor components, and 16S batch-run. Cluster 1: *Corynebacterium* dominant; Cluster 2: *Propionibacterium* dominant; Cluster 3: *Staphylococcus* dominant; Cluster 4: *Staphylococcus* and *Streptococcus* dominant; Cluster 5: unclassified *Neisseriaceae* dominant; and Cluster 6: *Moraxella* dominant

Overall, we identified 45 CpGs with differential methylation between at least two microbiome clusters (84 cluster contrasts; *p*_*Bonferroni*_ < 0.05, controlling for the number of CpG sites and pairwise contrasts of microbiome clusters). DMPs, effect sizes, *p*-values, and a summary of mapped genes are listed in Table 2; effect estimates and *p*-values for all cluster contrasts of each DMP are listed in Supplemental Table S2. We visualized the relationships between cluster assignment and DNAm levels using box plots (Fig. 4 and Supplemental Fig. S5). Absolute effect sizes ranged from 0.20 to 12.45% (Beta-value scale ⋅ 100) (median = 0.95%). Effect sizes for 23 CpGs (51%) were > 1%, the reported variation between technical replicates [41]. The greatest number of differentially methylated positions (DMPs) was associated with Cluster 3 (*Staphylococcus* dominant) compared to 6 (*Moraxella* dominant) (20 DMPs), followed by Cluster 2 (*Propionibacterium* dominant) compared to 6 (15 DMPs) and Cluster 2 compared to 3 (12 DMPs) (Fig. 3A). There was some overlap across individual CpGs. Namely, Cluster 1 compared to 2 and Cluster 2 compared to 3 shared 7 DMPs; Cluster 1 compared to 6, Cluster 2 compared to 6, and Cluster 3 compared to 6 shared 5 DMPs; and Cluster 1 compared to 6 and Cluster 3 compared to 6 shared 1 DMP (Fig. 3B).

**Table 2:** Differentially methylated positions associated with microbiome cluster contrasts. Results from robust linear models adjusted for sine and cosine of season to control for annual and biannual trends, age at sample collection, race and ethnicity, BMI *z*-score, maternal education, 10 ReFACTor components, and 16S batch-run. CpGs with absolute effect sizes > 1% are bolded. Cluster 1: *Corynebacterium* dominant; Cluster 2: *Propionibacterium* dominant; Cluster 3: *Staphylococcus* dominant; Cluster 4: *Staphylococcus* and *Streptococcus* dominant; Cluster 5: unclassified *Neisseriaceae* dominant; and Cluster 6: *Moraxella* dominant

CpG	Chr	Pos	Gene	Contrast	Difference in % methylation ^a^	*p*_Bonferroni_	Gene name and summary
cg16728516	1	1,342,558	*MRPL20*	Cluster 1 versus 6	− 0.20	0.002	Mitochondrial ribosomal protein; involved in protein synthesis in mitochondria
				Cluster 3 versus 6	− 0.20	0.015	
				Cluster 5 versus 6	− 0.21	0.030	
cg01074955	1	5,948,555	*NPHP4*	Cluster 2 versus 3	− 1.39	0.032	Nephrocystin 4; involved in ciliary trafficking and localizes to the transition zone of respiratory epithelial cells [42]
cg15700020	1	15,944,322	*DDI2*	Cluster 2 versus 6	− 1.18	0.001	DNA damage inducible 1 homolog 2; involved in protein degradation [43]
cg24592462	1	42,801,081	*FOXJ3*	Cluster 1 versus 6	− 0.25	0.041	Forkhead box j3; cell cycle regulator
				Cluster 2 versus 6	− 0.25	0.026	
				Cluster 3 versus 6	− 0.28	0.001	
				Cluster 4 versus 6	− 0.26	0.045	
				Cluster 5 versus 6	− 0.28	0.014	
cg00820581	1	174,969,299	*CACYBP*	Cluster 1 versus 6	− 1.51	0.026	Calcyclin binding protein; involved in protein degradation [44]
cg07850967	2	54,785,550	*SPTBN1*	Cluster 1 versus 6	− 0.40	1.09 × 10^−6^	Spectrin beta, non-erythrocytic 1; involved in regulation of mitochondrial respiratory function [45]
				Cluster 2 versus 6	− 0.37	3.76 × 10^−4^	
				Cluster 3 versus 6	− 0.37	3.05 × 10^−4^	
				Cluster 4 versus 6	− 0.38	0.001	
cg23699748	2	109,743,314		Cluster 1 versus 2	1.90	0.001	
				Cluster 2 versus 3	− 3.00	2.25 × 10^−5^	
cg18104979	2	234,077,733	*INPP5D*	Cluster 1 versus 2	1.14	1.48 × 10^−10^	Inositol polyphosphate-5-phosphatase D; involved in B-cell development and function
				Cluster 2 versus 3	− 1.89	1.41 × 10^−11^	
				Cluster 2 versus 4	− 1.90	1.07 × 10^−6^	
				Cluster 2 versus 5	− 1.64	0.002	
cg19565299	2	242,707,237	*D2HGDH*	Cluster 1 versus 2	0.51	0.019	D-2-hydroxyglutarate dehydrogenase; involved in mitochondrial metabolism [46]
				Cluster 2 versus 3	− 0.70	0.002	
cg01025283	3	138,327,728	*FAIM*	Cluster 2 versus 6	− 0.77	0.028	Fas apoptotic inhibitory molecule; inhibits apoptosis, regulates B-cell signaling and differentiation
cg19423735	4	184,366,198	*CDKN2AIP*	Cluster 2 versus 6	− 0.46	0.020	CDKN2A interacting protein; regulates response to DNA damage
				Cluster 3 versus 6	− 0.46	0.038	
cg05483076	5	74,347,539		Cluster 4 versus 6	10.15	0.003	–
cg06431905	5	148,931,119	*CSNK1A1*	Cluster 3 versus 6	− 0.21	0.023	Casein kinase 1 alpha 1; involved in signal transduction and kinase activity
cg16424683	5	177,541,051	*N4BP3*	Cluster 1 versus 6	− 0.74	0.026	NEDD4 binding protein 3; involved in innate immune antiviral response [47]
				Cluster 2 versus 6	− 0.80	0.001	
				Cluster 3 versus 6	− 0.82	0.001	
cg12221475	6	1,390,622	*FOXF2*	Cluster 2 versus 6	− 0.66	0.032	Forkhead box 2
cg08197824	7	12,479,882		Cluster 2 versus 3	− 1.36	0.006	–
cg19084794	8	96,086,565		Cluster 1 versus 6	12.45	1.99 × 10^−4^	–
cg05629953	8	133,493,136	*KCNQ3*	Cluster 3 versus 6	− 0.39	0.002	Potassium voltage-gated channel subfamily Q member 3; potassium channel subunit
cg22676654	8	133,787,679	*PHF20L1*	Cluster 3 versus 6	− 0.39	0.013	PHD finger protein 20 Like 1; regulates protein degradation
cg05579187	9	20,015,670		Cluster 1 versus 6	4.55	0.032	–
cg20659435	10	77,156,218		Cluster 2 versus 6	− 4.88	0.019	–
				Cluster 5 versus 6	− 5.16	0.035	
cg04892170	10	128,076,910	*ADAM12*	Cluster 2 versus 6	− 2.29	0.006	ADAM metallopeptidase domain 12; proteinase expressed in lung [48]
				Cluster 3 versus 6	− 2.22	0.047	
cg18238734	12	26,986,119	*ITPR2*	Cluster 3 versus 6	− 0.75	0.030	Inositol 1,4,5-trisphosphate receptor Type 2; regulates calcium homeostasis, associated with inflammation [49]
cg15620146	12	49,318,784	*FKBP11*	Cluster 1 versus 6	− 0.96	1.35 × 10^−5^	FKBP prolyl isomerase 11; antibody folding catalyst, increased expression in idiopathic pulmonary fibrosis
				Cluster 2 versus 6	− 1.04	1.40 × 10^−7^	
				Cluster 3 versus 6	− 1.00	8.30 × 10^−6^	
				Cluster 4 versus 6	− 0.92	0.005	
				Cluster 5 versus 6	− 1.03	1.66 × 10^−5^	
cg18567954	12	113,496,168	*DTX1*	Cluster 2 versus 3	− 1.97	0.030	Deltex E3 ubiquitin ligase 1; regulates the Notch signaling pathway
cg13474619	12	131,356,588	*RAN*	Cluster 2 versus 6	− 0.64	3.68 × 10^−5^	Ras-related nuclear protein; GTP binding protein
				Cluster 3 versus 6	− 0.59	0.003	
				Cluster 4 versus 6	− 0.59	0.012	
cg11510557	12	132,312,857	*MMP17*	Cluster 2 versus 6	− 0.41	0.002	Matrix metallopeptidase 17; involved in extracellular matrix breakdown, elevated in lung cancer cells [50]
				Cluster 3 versus 6	− 0.40	0.020	
cg16797691	13	95,363,755	*SOX21*	Cluster 3 versus 6	− 0.32	0.032	SRY-box transcription factor 21; involved in differentiation of airway epithelial cells [51]
cg04229722	13	107,190,457		Cluster 1 versus 2	1.05	7.03 × 10^−5^	–
				Cluster 2 versus 3	− 1.49	6.28 × 10^−5^	
cg15346917	14	103,801,136	*EIF5*	Cluster 3 versus 6	− 0.63	0.025	Eukaryotic translation initiation factor 5; involved in GTP hydrolysis and regulation of immune response [52]
cg19145592	15	25,511,348	*SNORD115-46*	Cluster 1 versus 2	1.08	6.76 × 10^−7^	Small nucleolar RNA, C/D Box 115 − 46
				Cluster 2 versus 3	− 1.59	1.37 × 10^−6^	
				Cluster 2 versus 5	− 1.58	0.023	
cg12665973	15	42,867,875	*STARD9*	Cluster 3 versus 6	− 0.49	0.007	StAR related lipid transfer domain containing 9
cg23456396	16	2,473,004	*ABCA17P*	Cluster 1 versus 2	2.67	0.008	ATP binding cassette subfamily a member 17, pseudogene
cg00624878	16	3,783,536	*CREBBP*	Cluster 1 versus 2	1.01	5.14 × 10^−12^	CREB binding protein; involved in epithelial barrier function [53]
				Cluster 2 versus 3	− 1.69	8.74 × 10^−13^	
				Cluster 2 versus 4	− 1.48	5.12 × 10^−5^	
				Cluster 2 versus 5	− 1.82	4.87 × 10^−8^	
cg05256656	16	67,290,583	*SLC9A5*	Cluster 2 versus 3	− 2.21	0.030	Solute carrier family 9 member A5; regulates intracellular pH
cg04202853	17	61,777,461	*LIMD2*	Cluster 3 versus 6	− 0.25	0.026	LIM domain containing 2; involved in actin filament binding
cg02701084	17	73,975,226	*ACOX1; C17orf106*	Cluster 1 versus 6	− 0.40	0.035	Acyl-CoA oxidase 1; involved in the fatty acid beta-oxidation pathway
				Cluster 2 versus 6	− 0.41	0.024	
				Cluster 3 versus 6	− 0.42	0.026	
cg23957800	19	1,918,247	*SCAMP4*	Cluster 1 versus 2	1.07	0.036	Secretory carrier membrane protein 4; involved in cellular senescence [54]
cg19385711	19	1,978,277	*CSNK1G2*	Cluster 2 versus 3	− 1.13	0.037	Casein kinase 1 gamma 2; involved in endocytosis and Wnt signaling
cg19254532	19	6,712,593	*C3*	Cluster 1 versus 2	0.40	0.006	Complement C3; involved in inflammation and possesses antimicrobial activity
				Cluster 2 versus 3	− 0.50	0.016	
cg01392841	19	47,016,869		Cluster 2 versus 5	− 1.30	0.002	–
				Cluster 3 versus 5	− 1.24	0.019	
cg13801271	19	47,017,048		Cluster 1 versus 5	− 2.22	0.031	–
				Cluster 2 versus 5	− 2.60	5.63 × 10^−6^	
				Cluster 3 versus 5	− 2.46	0.001	
cg26791489	20	57,463,330	*GNAS*	Cluster 2 versus 6	− 6.18	0.018	GNAS complex locus; involved in signal transduction
				Cluster 3 versus 6	− 6.13	0.046	
cg14815005	22	22,222,162	*MAPK1*	Cluster 2 versus 6	− 0.93	0.001	Mitogen-activated protein kinase 1; anti-inflammatory protein
cg15641348	22	41,985,832	*PMM1*	Cluster 3 versus 6	− 0.35	0.018	Phosphomannomutase 1; involved in N-linked glycosylation

^a^Difference in % methylation, i.e., Beta-value (DNA methylation proportion) × 100, derived using the M-mean-M-model method [32]

The individual DMPs with the greatest number of significant contrasts (*p*_*Bonferroni*_ < 0.05) were cg24592462, annotated to *FOXJ3*, and cg15620146, annotated to *FKBP11*, both of which were hypermethylated in Cluster 6 (*Moraxella* dominant) compared to all other clusters (Fig. 4A, B). Several DMPs were hypomethylated in Cluster 2 (*Propionibacterium* dominant) compared to other clusters. For example, cg00624878, annotated to *CREBBP*, had lower methylation in Cluster 2 compared to Clusters 1, 3, 4, and 5 (*p*_*Bonferroni*_ < 0.05); Cluster 2 compared to 6 was suggestively significant (*p* = 3.35 × 10^−6^) (Fig. 4C). Similarly, cg18104979, annotated to *INPP4D*, had lower methylation in Cluster 2 compared to Clusters 1, 3, 4, and 5 (*p*_*Bonferroni*_ < 0.05); Cluster 2 compared to 6 was suggestively significant (*p* = 2.26 × 10^−5^) (Fig. 4D).

**Fig. 5:**
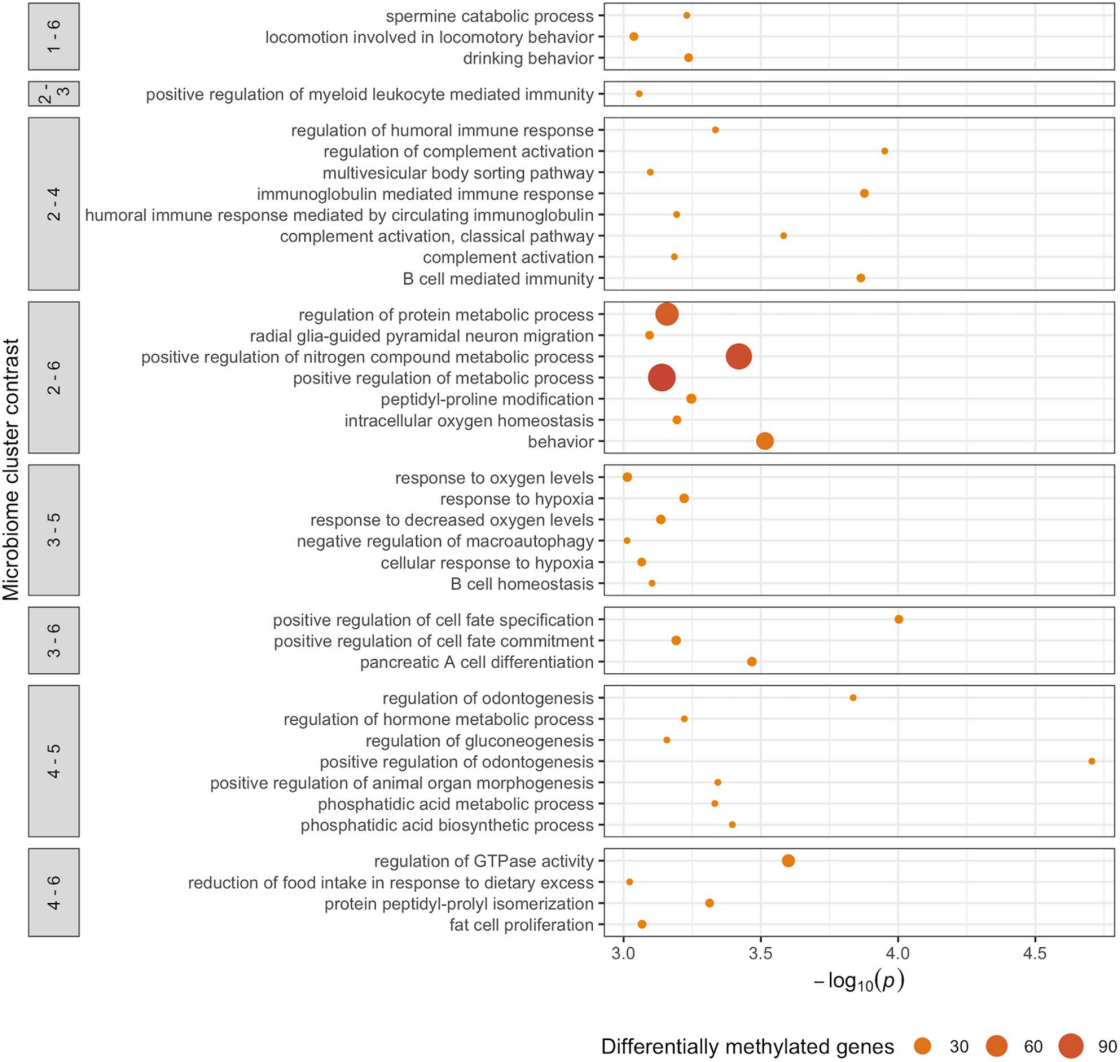
Gene Ontology (GO) Biological Pathway (BP) terms enriched for differentially methylated genes associated with microbiome cluster contrasts (*FDR* < 0.05). Pathways with > 1 differentially methylated gene and *p* < 0.001 are shown. Cluster 1: *Corynebacterium* dominant; Cluster 2: *Propionibacterium* dominant; Cluster 3: *Staphylococcus* dominant; Cluster 4: *Staphylococcus* and *Streptococcus* dominant; Cluster 5: unclassified *Neisseriaceae* dominant; and Cluster 6: *Moraxella* dominant

We conducted GO [33, 34] enrichment analysis using CpGs associated with microbiome clusters at an FDR adjusted *p*-value < 0.05. Biological Pathway GO terms with > 1 differentially methylated gene and *p* < 0.001 are shown in Fig. 5; all GO pathways with > 1 differentially methylated gene and *p* < 0.05 are included in Supplemental Table S3. Of note, we found enrichment of pathways involving myeloid leukocyte mediated immunity (Cluster 2 vs. 3), humoral immune response, immunoglobulin mediated immune response, complement activation (involved in killing microbes), B-cell mediated immunity (Cluster 2 vs. 6), response to hypoxia and oxygen levels, B-cell homeostasis (Cluster 3 vs. 5), and phosphatidic acid metabolic and biosynthetic processes (Cluster 4 vs. 5).

We found evidence that some DMPs may be affected by meQTLs. Most notably, cg13801271 (intergenic, chromosome 19) had higher mean methylation levels in Cluster 5 (unclassified *Neisseriaceae* dominant) versus Clusters 1, 2, and 3 (*p*_*Bonferroni*_ < 0.05). Beta-values at this CpG ranged from 0.01 to 0.41 and displayed a bimodal distribution (Supplemental Fig. S6). Using K-means, we clustered Beta-values into two groups centered at 0.02 and 0.22, and the proportion of samples assigned to each CpG group was significantly different between microbiome clusters (Chi-squared *p* = 0.001). cg13801271 and a CpG two base pairs downstream, cg15825916, have previously been associated with *cis*-meQTLs in blood; [37] cg13801271 has also been identified as part of a group of CpGs under meQTL influence (including the DMP cg01392841 and cg15825916) [55]. Nine other DMPs have also previously been associated with meQTLs in blood (Supplemental Table S4).

### Bacterial genera abundance is associated with DNA methylation

For each of the 45 microbiome cluster-associated DMPs, we analyzed associations of individual bacterial genera abundance and DNAm levels. Adjusted models were conducted using ANCOM-BC2 [38] and included 39 taxa. A total of 6 taxa were associated with DNAm (*FDR* < 0.05, controlling for the number taxa) and passed sensitivity analyses for pseudo-counts as shown in Table 3. Taxa associated with DNAm at *FDR* < 0.05 are shown in Supplemental Table S5 and results of all analyses are shown in Supplemental Table S6.

**Table 3:** Associations of taxa abundance with DNA methylation. Results from ANCOM-BC2 analysis of the 45 differentially methylated positions associated with microbiome cluster contrasts. Models were adjusted for sine and cosine of season to control for annual and biannual trends, age at sample collection, race and ethnicity, BMI *z*-score, maternal education, 10 ReFACTor components, and 16S batch-run. Taxa associated with DNA methylation at *FDR* < 0.05 and that passed sensitivity analyses are shown. Cluster 1: *Corynebacterium* dominant; Cluster 2: *Propionibacterium* dominant; Cluster 3: *Staphylococcus* dominant; Cluster 4: *Staphylococcus* and *Streptococcus* dominant; Cluster 5: unclassified *Neisseriaceae* dominant; and Cluster 6: *Moraxella* dominant

Genus	CpG	Chr	Pos	Gene	logFC per IQR M-value	*p*	FDR	CpG association in cluster EWAS
*Propionibacterium*	cg01074955	1	5,948,555	*NPHP4*	− 1.37	5.12 × 10^−11^	2.00 × 10^−9^	Cluster 2 versus 3
*Unclassified Actinomycetales*					− 1.01	5.46 × 10^−5^	0.001	
*Corynebacterium*					− 0.68	0.001	0.006	
*Peptoniphilus*					− 0.83	0.001	0.006	
*Anaerococcus*					− 0.71	0.002	0.014	
*Propionibacterium*	cg23699748	2	109,743,314		− 1.70	1.72 × 10^−12^	6.70 × 10^−11^	Cluster 1 versus 2; Cluster 2 versus 3
*Propionibacterium*	cg18104979	2	234,077,733	*INPP5D*	− 1.62	5.02 × 10^−13^	1.96 × 10^−11^	Cluster 1 versus 2; Cluster 2 versus 3; Cluster 2 versus 4; Cluster 2 versus 5
*Propionibacterium*	cg19565299	2	242,707,237	*D2HGDH*	− 1.10	3.78 × 10^−5^	0.001	Cluster 1 versus 2; Cluster 2 versus 3
*Propionibacterium*	cg08197824	7	12,479,882		− 0.88	0.001	0.041	Cluster 2 versus 3
*Moraxella*	cg04892170	10	128,076,910	*ADAM12*	0.64	0.001	0.029	Cluster 2 versus 6; Cluster 3 versus 6
*Propionibacterium*	cg18567954	12	113,496,168	*DTX1*	− 1.04	0.000	0.009	Cluster 2 versus 3
*Propionibacterium*	cg04229722	13	107,190,457		− 1.08	1.29 × 10^−5^	0.001	Cluster 1 versus 2; Cluster 2 versus 3
*Propionibacterium*	cg19145592	15	25,511,348	*SNORD115-46*	− 0.77	8.42 × 10^−7^	3.28 × 10^−5^	Cluster 1 versus 2; Cluster 2 versus 3; Cluster 2 versus 5
*Peptoniphilus*					− 0.46	0.002	0.020	
*Propionibacterium*	cg23456396	16	2,473,004	*ABCA17P*	− 0.81	0.000	0.011	Cluster 1 versus 2
*Propionibacterium*	cg00624878	16	3,783,536	*CREBBP*	− 1.20	1.85 × 10^−9^	7.23 × 10^−8^	Cluster 1 versus 2; Cluster 2 versus 3; Cluster 2 versus 4; Cluster 2 versus 5
*Propionibacterium*	cg05256656	16	67,290,583	*SLC9A5*	− 1.17	2.19E-07	8.52 × 10^−6^	Cluster 2 versus 3
*Peptoniphilus*					− 0.81	0.001	0.022	
*Propionibacterium*	cg23957800	19	1,918,247	*SCAMP4*	− 1.08	2.45 × 10^−5^	0.001	Cluster 1 versus 2
*Propionibacterium*	cg19254532	19	6,712,593	*C3*	− 1.06	2.24 × 10^−5^	0.001	Cluster 1 versus 2; Cluster 2 versus 3
*Anaerococcus*	cg13801271	19	47,017,048		− 0.17	0.005	0.024	

**Fig. 6:**
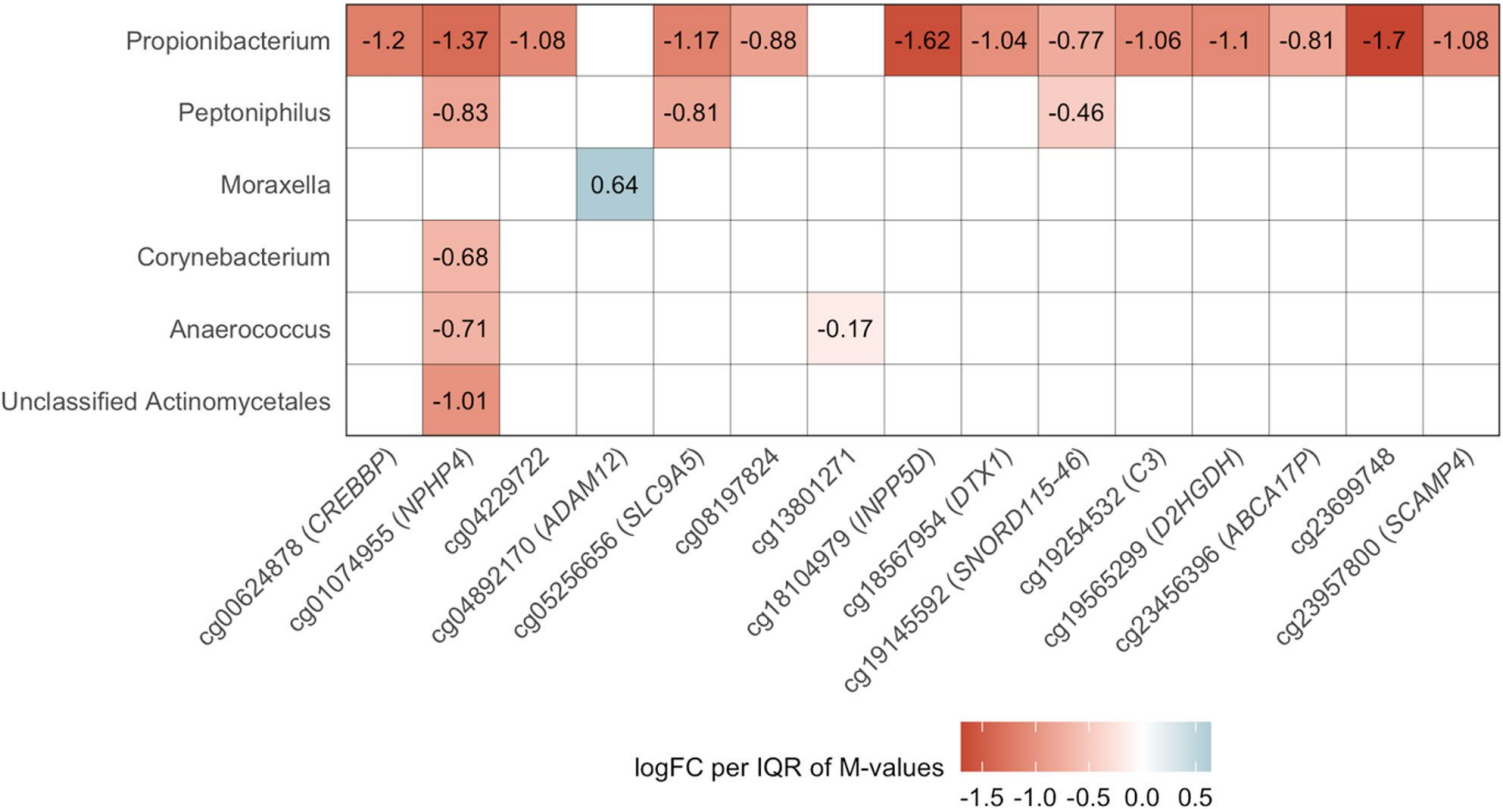
Associations of taxa abundance with DNA methylation. Results from ANCOM-BC2 analysis differentially methylated positions associated with microbiome cluster contrasts. Models were adjusted for sine and cosine of season to control for annual and biannual trends, age at sample collection, race and ethnicity, BMI *z*-score, maternal education, 10 ReFACTor components, and 16S batch-run. Associations with *FDR* < 0.05 and that passed sensitivity analyses are shown

*Propionibacterium* abundance was associated with DNAm at 13 CpGs: cg01074955 (*NPHP4*), cg23699748 (intergenic), cg18104979 (*INPP5D*), cg19565299 (*D2HGDH*), cg08197824 (intergenic), cg18567954 (*DTX1*), cg04229722 (intergenic), cg19145592 (*SNORD115-46*), cg23456396 (*ABCA17P*), cg00624878 (*CREBBP*), cg05256656 (*SLC9A5*), cg23957800 (*SCAMP4*), and cg19254532 (*C3*) (Fig. 6), all of which had a negative direction of association, i.e., greater *Propionibacterium* abundance was associated with lower DNAm levels. These *Propionibacterium*-associated CpGs all had lower levels of DNAm in Cluster 2 (*Propionibacterium* dominant) compared to other clusters in EWAS. Greater *Moraxella* abundance was positively associated with DNAm at one CpGs (cg04892170 (*ADAM12*)), which had greater mean methylation levels in Cluster 6 (*Moraxella* dominant) compared to Clusters 2 and 3 in the EWAS. In addition, *Peptoniphilus* abundance was associated with 2 CpGs (cg01074955 (*NPHP4*) and cg19145592 (*SNORD115-46*)), *Corynebacterium* abundance was associated with 1 CpG (cg01074955 (*NPHP4*)), *Anaerococcus* abundance was associated with 2 CpGs (cg01074955 (*NPHP4*) and cg13801271 (intergenic)), and unclassified *Actinomycetales* abundance was associated with 1 CpG (cg01074955 (*NPHP4*)).

### Epigenetic aging is associated with bacterial genera abundance

Nasal Horvath pan-tissue epigenetic age was weakly but significantly correlated with chronological age (*r* = 0.19; *p* < 0.001) and had a median absolute error (MAE) of 3.5 years. The strength of correlation was limited by a narrow range of chronological age among study participants (11.9–15.4 years). In adjusted linear models, Horvath EAD was not significantly associated with any cluster contrast (*p* > 0.05) (Supplemental Table S7). However, in differential abundance analysis, we found a trend towards a negative association of microbial abundance with Horvath EAD (Fig. S7). *Corynebacterium* abundance was associated with lower EAD at an FDR threshold and passed sensitivity analyses (logFC = − 0.14 per year increase in EAD; *FDR* = 0.046). Results for taxa with FDR < 0.05 are shown in Supplemental Table S8.

### Sensitivity analyses

We performed sensitivity analyses testing for associations of microbiome cluster assignment with DNAm controlling for asthma medication use during the past year. Overall, results were similar to our primary EWAS (Supplemental Table S9). We found 43 CpGs that were significantly associated with at least one microbiome cluster contrast (*p*_*Bonferroni*_ < 0.05), of which 39 were also identified in our primary analysis. The CpGs that were unique to our sensitivity analysis were cg09313188 (*BAT2*), cg12757684 (*PLAGL1*; *HYMAI*), cg01055561 (*VPS37C*), and cg20823662 (intergenic). Among the 84 significant cluster contrasts in our primary analyses, 17 contrasts representing 14 CpGs failed to reach Bonferroni-significance in our sensitivity analysis. However all had nominal *p*-values < 10^−6^ (Supplemental Table S9).

To evaluate the influence of outliers, we conducted sensitivity analyses by performing EWAS on winsorized DNAm data. Although we identified a smaller number of Bonferroni-significant DMPs, overall, results were similar to our primary analyses (Supplemental Table S10). Among 34 CpGs associated with microbiome clusters (*p*_*Bonferroni*_ < 0.05), 26 were identified in our primary analyses. The 8 CpGs unique to the sensitivity analyses were cg06861375 (ZNF697), cg10101468 (*B3GNT6*), cg08103551 (*CAPN5*), cg11582017 (*FURIN*), cg20823662 (intergenic), cg03821543 (*GNAS*), cg26161148 (*HRAT92*; *PDGFA*), and cg17368874 (*RECQL4*). A total of 34 cluster contrasts were found in our primary analyses but were not Bonferroni-significant in our sensitivity analysis; however, all had nominal *p*-values < 10^−5^ (Supplemental Table S10). It should be noted that cg24592462, hypermethylated in Cluster 6 (*Moraxella* dominant) compared to all other clusters in our main analyses (Fig. 4A), remained significantly associated with Cluster 6 compared to Cluster 3, but was no longer associated with the other cluster contrasts. In contrast, cg15620146 (Fig. 4B) remained significantly associated with Cluster 6 compared to Clusters 1, 2, 3, 4, and 5 (*p*_*Bonferroni*_ < 0.05).

Permutation analysis of EWAS was conducted with random cluster assignments. The distribution of DMPs (*p*_*Bonferroni*_ < 0.05) from permuted datasets is shown in Supplemental Fig. S8. Results showed that our observed EWAS signal of 45 DMPs exceeded that expected under the null hypothesis of no association between microbiome clusters and DNAm (empirical *p*-value = 0.006).

## Discussion

In this study, we aimed to provide insights into the interplay between the nasal microbiome and nasal epigenome. Associations of the nasal microbiome and epigenome with respiratory health have previously been studied separately in this cohort [17, 18]; the current analyses may help to provide insights to the relationship between two ‘omics layers that are important to respiratory health. In our sample of adolescents, we found that microbiome samples clustered into six distinct clusters defined on the genus level: *Corynebacterium* dominant, *Propionibacterium* dominant, *Staphylococcus* dominant, *Staphylococcus* and *Streptococcus* dominant, unclassified *Neisseriaceae* dominant, and *Moraxella* dominant. Forty-five CpG sites had differential methylation levels between two or more microbiome clusters. There was evidence that the abundance of individual taxa was driving some associations: the abundance of *Propionibacterium Peptoniphilus*, *Moraxella*, *Corynebacterium*, *Anaerococcus*, and unclassified *Actinomycetales* was associated with DNAm of at least one differentially methylated CpG. In addition, we found that greater abundance of *Corynebacterium* was associated with lower nasal epigenetic age deviation.

Previous studies have sought to characterize the nasal microbiome in children and adults; however, it is difficult to directly compare results to our study due to differences in ages, disease phenotypes, location of sample collection within the nasal cavity, and taxonomic levels reported. In a prospective study of infants (*N* = 923), *Staphylococcus* spp. and *Corynebacteriaceae* abundance decreased and *Moraxella* abundance increased from ages 2 months to 24 months [56]. However, this age-related pattern in dominant microbiota may reverse later in childhood and into adulthood. In a study of nasal swabs collected in healthy children (*N* = 30; mean age = 5 years) and adults (*N* = 24; mean age = 25 years), *Moraxella* had the greatest abundance in children, but represented less than 1% of the microbiome in adults [57]. A separate study of children (*N* = 9; mean age = 9.7 years) and adults (*N* = 10; mean age = 46 years) with chronic rhinosinusitis (*N* = 19) found that *Corynebacterium* had significantly higher abundance among adults [58]. Most similar to our study, the Human Microbiome Project identified four community classes of the microbiome of anterior nares sampled in adults (*N* = 236), characterized by *Corynebacterium*, *Propionibacterium*, *Staphylococcus*, and *Moraxella*, with *Moraxella* being the least common [59]. In our study of adolescents (mean age = 13 years), most samples were *Corynebacterium* (32%), *Propionibacterium* (22%) or *Staphylococcus* dominant (21%); whereas only 4% of samples were classified as *Moraxella* dominant. We also identified two additional clusters, *Staphylococcus* and *Streptococcus* dominant (11%) and unclassified *Neisseriaceae* dominant (9%), which had the greatest alpha diversity. This suggests that adolescents in our cohort may have been undergoing maturation from a childhood to adult nasal microbiome profile or that some microbiome clusters represented a state of dysbiosis, allowing us a unique opportunity to study the microbiome during this dynamic period.

Microbial diversity and abundance have been associated with respiratory health in children and adolescents, including in the current study population [18, 60]. It is hypothesized that microbiome-associated immune responses may be mediated in part by the epigenome [6, 19]. In a study conducted in the Copenhagen Prospective Studies on Asthma in Childhood (COPSAC) 2010 birth cohort (*N* = 468), the relationship between the upper airway microbiome (profiled from hypopharyngeal samples) in infancy and the epigenome (measured from inferior turbinate epithelial cell scrapings) and allergic rhinitis at age 6 was studied [61]. Decreased microbiome diversity at one week after birth was associated with allergic rhinitis in childhood, and, in mediation analysis, approximately 60% of the effect of the infant microbiome diversity on childhood allergic rhinitis was mediated through variation in DNAm. Among infants with severe bronchiolitis, the nasopharyngeal microbiome has been associated with differential DNAm in blood, and variation in DNAm was linked to expression of proteins related to immune pathways [62].

We identified 45 CpG sites with differential methylation levels between two or more microbiome clusters. These CpGs did not overlap with loci previously associated with asthma and airway inflammation in Project Viva [17]; however, the *Staphylococcus*-dominant Cluster (Cluster 2) was previously associated with aeroallergen sensitization in the current cohort [18]. The *Moraxella*-dominant Cluster (Cluster 6) had the most distinct epigenome profile and the greatest number of DMPs compared to other clusters. Compared to the *Propionibacterium-* and *Staphylococcus*-dominant Clusters (Clusters 2 and 3, respectively), the *Moraxella*-dominant Cluster had differential methylation at 10 common CpGs, which included CpGs mapped to genes related to lung function and cancer (*ADAM12*, *MMP17*, *FKBP11*, and *GNAS*) (summarized in Table 2). Additionally, genes with differential methylation in the *Moraxella*-dominant Cluster were related to inflammation, asthma (*ITPR2*, and *MAPK1*), and mitochondrial function (*MRPL20* and *SPTBN1*). Mitochondrial dysfunction has been linked to a variety of respiratory illnesses, including asthma, due to mitochondria’s role in mucus secretion, senescence, and immune cell response [63]. We also found DMPs (*N* = 43) associated with the *Propionibacterium*-dominant Cluster (Cluster 2), including 12 DMPs when compared to the *Staphylococcus*-dominant Cluster (Cluster 3). Twelve of these CpGs were also associated with *Propionibacterium* abundance, suggesting that many of the associations were driven by this taxon, rather than the overall microbiome profile. *Propionibacterium*-associated CpGs were mapped to genes with potential roles in cellular senescence (*SCAMP4*), inflammation, and antimicrobial activity (C3).

Interpretation of results should also consider effect sizes. Twenty-three CpGs with differential methylation (51%) had absolute effect sizes > 1%, the reported variation between technical replicates [41]. We also identified differences in methylation levels between clusters as high as 10% (cg05483076) and 12% (cg19084794). In Project Viva, top loci in an EWAS of nasal DNAm and asthma had absolute effect sizes between 0.48 and 6.09% [17], suggesting that the majority of our DMPs had methylation differences comparable to those associated with respiratory health outcomes. However, 22 (49%) of DMPs had absolute effect sizes ≤ 1%, which should be cautiously interpreted.

Among CpGs associated with microbiome clusters, we identified several as putatively related to meQTLs due to their distribution of Beta-values and previously reported associations with genetic variants [37]. Particularly, cg13801271 and cg01392841 (chromosome 19, 5 kb upstream of the pseudogene *PPP5D1P*) were two neighboring CpGs differentially methylated in the unclassified *Neisseriaceae*-dominant Cluster (Cluster 5). Although these associations may be a result of bias due to an unbalanced distribution of SNPs across clusters, underlying genetic variation may also affect the host environment and suitability for specific taxa. The proportion of DMPs that have putative meQTLs in our study (22.2%) was less than the proportion of meQTL-related CpGs in blood measured on the MethylationEPIC array reported in the EPIGEN MeQTL Database (34.2%) [37], suggesting that meQTLs are not overrepresented in our results. Furthermore, genome-wide association studies have found significant associations of genetic variation with the nasal [64, 65] and oral [66, 67] microbiome. Future research may uncover links between genetic and epigenetic regulation, gene expression, and microbiome composition.

We also investigated associations of microbiome clusters and taxon abundance with Horvath pan-tissue EAD. The Horvath pan-tissue clock is a well-established biomarker developed to estimate age across human tissues and across the life course [39]. EAD, or the difference between epigenetic age and chronological age, is a measure of biological aging associated with the risk of mortality and age-related morbidities [68], but has also been proposed as a biomarker of asthma and allergic diseases [69]. Beyond capturing intrinsic cellular age-related changes, epigenetic clocks, including the Horvath pan-tissue clock, consistently estimate younger ages for naïve compared to activated cells and are reflective of immune cell proportions [70]. Increases in activated T cells characteristic of allergic asthma [71] may therefore be captured by changes in epigenetic aging. Supporting this hypothesis, in the current cohort, serum IgE levels and IgE sensitization have been positively associated with EAD in blood [72] and asthma, FeNO (a biomarker of allergic disease), IgE, and IgE sensitization have been positively associated with EAD in nasal samples [17]. Although EAD did not differ significantly between microbiome clusters, we found that *Corynebacterium* abundance was associated with lower Horvath EAD. *Corynebacterium* includes species that may protect against respiratory infections [73] and have been associated with asthma control in children [74]. Therefore, our results suggest that *Corynebacterium* supports respiratory health through commensal interactions with the host immune system.

Our study was strengthened by the microbiome and epigenome measured in the same samples collected from nasal anterior nares. Although we did not have samples from other upper or lower respiratory tract tissues, collection of nasal biospecimens requires sampling techniques that can be applied in diverse research and clinical settings. We utilized multiple analysis techniques, including conducting a microbiome cluster analysis, epigenome-wide association study, and differential abundance analysis, which allowed us to investigate the relationship between microbiome clusters and taxa abundance with DNAm levels. Our study’s strengths also included its relatively large sample size, which increased our power to detect small effect sizes. However, it should be noted that some microbiome clusters, particularly Cluster 6, were comprised of a small number of samples. To evaluate if outliers could be driving associations, we conducted sensitivity analyses with winsorized DNAm. Results were largely consistent with our primary analyses but did suggest that some observed associations could be driven by outliers in Cluster 6.

We also had several limitations that should be noted. First, our data was cross-sectional. Both the microbiome and epigenome are dynamic, and future studies should evaluate how longitudinal changes in the microbiome may affect DNA methylation signatures and vice versa. We were also unable to evaluate the causal direction of association between observed associations. The microbiome may be influenced by the host environment and regulation of the nasal mucosa, and biologic factors secreted by microbes may influence epigenetic markers, particularly those related to immune function. Deciphering the direction of association is important to inform clinical interventions to address respiratory illnesses. Second, although loss to follow-up could impact inclusion of participants in our study, we do not believe that available data would bias results. Results may also not be generalizable to populations, including children with different social and environmental exposures or representing more diverse ancestries. Third, our results may be impacted by unadjusted confounders, such as vaccinations, medication use, or illness. However, sensitivity analyses adjusting for asthma medication use yielded consistent results. Nasal swabs were not collected from children with symptoms of acute nasal illness. While some misclassification of allergy symptoms versus acute illness may have occurred during sample collection, one of the objectives of the parent study was to compare having versus not having asthma/allergies, and we did not want to bias the sample by excluding children who might have chronic allergy symptoms. Fourth, 16S rRNA sequencing processed as OTUs does not provide species-level information, and we were unable to test if species abundance is associated with DNAm. Finally, our differential abundance analysis was limited to evaluating associations of microbial abundance with DNAm at CpGs identified in our cluster EWAS, and focusing on major sources of variability in the microbiome may mask associations with low-abundance genera or genera that varied within clusters. Our approach did not test for associations of all genera with all CpGs, as this would result in 51 genera ⋅ 715,023 CpGs = 36,466,173 tests. Studies with larger sample sizes and increased power may have the ability to conduct microbiome ⋅ epigenome-wide analyses to uncover novel associations of genera abundance with methylation levels. Future studies may also take alternate approaches to multiomics data integration, which reveal novel interactions between the microbiome and epigenome.

## Conclusions

In summary, our study supports the hypothesis that there is a statistically detectable association between the nasal microbiome and nasal epigenome, although the biological implications of these effects warrant further investigation. We identified 6 distinct microbiome clusters with genus-level profiles consistent with previous studies (*Corynebacterium* dominant, *Propionibacterium* dominant, *Staphylococcus* dominant, *Staphylococcus* and *Streptococcus* dominant, unclassified *Neisseriaceae* dominant, and *Moraxella* dominant). CpG sites with differential methylation between two or more microbiome clusters were mapped to genes related to asthma, lung cancer and function, mitochondrial function, inflammation, and immune function. Taxa abundance, particularly *Propionibacterium*, was also associated with differential DNAm, suggesting that some associations were driven by individual genera. Furthermore, we found that *Corynebacterium* abundance was associated with lower epigenetic age deviation, which may be due to immune-driven changes in epigenetic age. Overall, our findings contribute to the growing body of literature linking the microbiome to other omics layers. Future research may seek to understand how microbiome-epigenome interactions affect respiratory health, particularly in early life and adolescence, periods of maturation and transition of the microbiome.

## Supplementary Information

Below is the link to the electronic supplementary material.

Supplementary Material 1.

Supplementary Material 2.

Supplementary Material 3.

## Data Availability

Datasets analyzed in this study are not publicly available because consent for public release of microbiome and epigenetic data not obtained from participants. However, data and code to generate figures and tables are available with the appropriate permission from the Project Viva study team and investigators upon reasonable request and Institutional Review Board approval. The formal protocol for investigators seeking to use Project Viva data is available at [https://www.projectviva.org](https:/www.projectviva.org) . To facilitate data sharing, Project Viva has developed a web-based research portal, the Research Operations and Data Management Platform or Viva ROADMaP (vivaroadmap.net), through which investigators can access information and documentation related to the data, propose analytical plans, and submit dataset requests. For more information, investigators can contact the Project Viva Principal Investigators, Emily Oken and Marie-France Hivert, at [project\_viva@hphci.harvard.edu](mailto: project_viva@hphci.harvard.edu) . Detailed information about Project Viva cohort, study design, and data can be found in the published cohort profile and updates [20–22].

## Abbreviations

DNAm: DNA methylation
STORMS: Strengthening the organization and reporting of microbiome studies
BMI: Body mass index
rRNA: Ribosomal RNA
OTUs: Operational taxonomic units
PCs: Principal components
MAF: Minor allele frequency
ReFACTor: Reference-free adjustment for cell-type composition
KL: Kullback-Leibler
JSD: Jensen-Shannon divergence
CHI: Calinski-Harabasz index
PAM: Partitioning around medoids
ARI: Adjusted rand index
SD: Standard deviation
EWAS: Epigenome-wide association study
BIF: Bayesian inflation factor
GO: Gene Ontology
FDR: False discovery rate
DMPs: Differentially methylated positions
mQTLs: Methylation quantitative trait loci
ANCOM-BC2: Analysis of compositions of microbiomes with bias correction 2
logFC: log(fold change)
EAD: Epigenetic age deviation
RA: Relative abundance
MAE: Cedian absolute error
COSPAC: Copenhagen prospective studies on asthma in childhood
