# Supplementary material for: Interplay of the nasal microbiome and epigenome among adolescents
Source: Clin Epigenetics. 2026 Feb 27;18:93. doi: 10.1186/s13148-026-02093-1 (PMC13202811; doi:10.1186/s13148-026-02093-1)

**Supplemental Material**

**Title:** Interplay of the nasal microbiome and epigenome among adolescents

**Authors:** Anne K. Bozack, Javier Perez-Garcia, Sheryl Rifas-Shiman, Yanjiao Zhou, Joanne Sordillo, Jenny Jyoung Lee, Brent Coull, Peggy S. Lai, Emily Oken, Marie-France Hivert, Diane R. Gold, Andres Cardenas

## Supplementary Table S1: STORMS checklist.

| Number | Item | Recommendation | Item Source | Additional Guidance | Yes/No/ NA | Comments or location in manuscript |
|---|---|---|---|---|---|---|
| **Abstract** | | | | | | |
| 1.0 | Structured or Unstructured Abstract | Abstract should include information on background, methods, results, and conclusions in structured or unstructured format. | STORMS | | Yes | Lines 2-24 |
| 1.1 | Study Design | State study design in abstract. | STORMS | See 3.0 for additional information on study design. | Yes | Line 5 |
| 1.2 | Sequencing methods | State the strategy used for metagenomic classification. | STORMS | For example, targeted 16S by qPCR or sequencing, shotgun metagenomics, metatranscriptomics, etc. | Yes | Line 7 |
| 1.3 | Specimens | Describe body site(s) studied. | STORMS | | | Line 6 |
| **Introduction** | | | | | | |
| 2.0 | Background and Rationale | Summarize the underlying background, scientific evidence, or theory driving the current hypothesis as well as the study objectives. | STORMS | | Yes | Lines 28-61 |
| 2.1 | Hypotheses | State the pre-specified hypothesis. If the study is exploratory, state any pre-specified study objectives. | STORMS | | Yes | Lines 64-70 |
| **Methods** | | | | | | |
| 3.0 | Study Design | Describe the study design. | STORMS | Observational (Case-Control, Cohort, Cross-sectional survey, etc.) or Experimental (Randomized controlled trial, Non-randomized controlled trial, etc.). For a brief description of common study designs see: DOI: 10.11613/BM.2014.022<br>If applicable, describe any blinding (e.g. single or double-blinding) used in the course of the study. | Yes | Lines 74-76 |
| 3.1 | Participants | State what the population of interest is, and the method by which participants are sampled from that population. Include relevant information on physiological state of the subjects or stage in the life history of disease under study when participants were sampled. | STORMS | Examples of the population of interest could be: adults with no chronic health conditions, adults with type II diabetes, newborns, etc. This is the total population to whom the study is hoped to be generalizable to. The sampling method describes how potential participants were selected from that population.<br>If the participants are from a substudy of a larger study, provide a brief description of that study and cite that study.<br>Clearly state how cases and controls are defined.<br>An example of relevant physiological state might be pre/post menopausal for a vaginal microbiome study; examples of stage in the life history of disease could be whether specimens were collected during active or dormant disease, or before or after treatment. | Yes | Lines 74-79 |
| 3.2 | Geographic location | State the geographic region(s) where participants were sampled from. | MIxS: geographic | Geographic coordinates can be reported to prevent potential ambiguities if necessary. | Yes | Line 78 |

| | | | | | | |
|---|---|---|---|---|---|---|
| | | | location (country and/or sea,region) | | | |
| 3.3 | Relevant Dates | State the start and end dates for recruitment, follow-up, and data collection. | STORMS | Recruitment is the period in which participants are recruited for the study. In longitudinal studies, follow-up is the date range in which participants are asked to complete a specific assessment. Finally, data collection is the total period in which data is being collected from participants including during initial recruitment through all follow-ups. | Yes | Lines 75-81 |
| 3.4 | Eligibility criteria | List any criteria for inclusion and exclusion of recruited participants. | Modified STROBE | Among potential recruited participants, how were some chosen and others not? This could include criteria such as sex, diet, age, health status, or BMI.<br>If there is a primary and validation sample, describe inclusion/exclusion criteria for each. | Yes | Lines 78-79 |
| 3.5 | Antibiotics Usage | List what is known about antibiotics usage before or during sample collection. | STORMS | If participants were excluded due to current or recent antibiotics usage, state this here. Other factors (e.g. proton pump inhibitors, probiotics, etc.) that may influence the microbiome should also be described as well. | Yes | Lines 485-487 |
| 3.6 | Analytic sample size | Explain how the final analytic sample size was calculated, including the number of cases and controls if relevant, and reasons for dropout at each stage of the study. This should include the number of individuals in whom microbiome sequencing was attempted and the number in whom microbiome sequencing was successful. | STORMS | Consider use of a flow diagram (see template at https://stormsmicrobiome.org/figures). Also state sample size in abstract.<br>If power analysis was used to calculate sample size, describe those calculations. | Yes | Supplemental Figure S1 |
| 3.7 | Longitudinal Studies | For longitudinal studies, state how many follow-ups were conducted, describe sample size at follow-up by group or condition, and discuss any loss to follow-up. | STORMS | If there is loss to follow-up, discuss the likelihood that drop-out is associated with exposures, treatments, or outcomes of interest. | NA | |
| 3.8 | Matching | For matched studies, give matching criteria. | Modified STROBE | "Matched" refers to matching between comparable study participants as cases and controls or exposed / unexposed.<br>Indicate whether participants were individual or frequency matched and in what ratio were they matched (e.g. 1 case to 1 control). | NA | |
| 3.9 | Ethics | State the name of the institutional review board that approved the study and protocols, protocol number and date of approval, and procedures for obtaining informed consent from participants. | STORMS | | Yes | Lines 83-84 |
| 4.0 | Laboratory methods | State the laboratory/center where laboratory work was done. | STORMS | Provide a reference to complete lab protocols if previously published elsewhere such as on protocols.io. Note any modifications of lab protocols and the reason for protocol modifications. | Yes | Line 109-110 |
| 4.1 | Specimen collection | State the body site(s) sampled from and how specimens were collected. | MIxS: sample collection device or method; host body site | Use terms from the Uber-anatomy Ontology (https://www.ebi.ac.uk/ols/ontologies/uberon) to describe body sites in a standardized format. | Yes | Lines 98-99 |
| 4.2 | Shipping | Describe how samples were stored and shipped to the laboratory. | STORMS | Include length of time from collection to receipt by the lab and if temperature control was used during shipping. | Yes | Lines 103-105 |
| 4.3 | Storage | Describe how the laboratory stored samples, including time between collection and storage and any preservation buffers or refrigeration used. | STORMS | State where each procedure or lot of samples was done if not all in the same place. Include reagent/lot/catalogue #s for storage buffers. | Yes | Line 103-105 |
| 4.4 | DNA extraction | Provide DNA extraction method, including kit and version if relevant. | MIxS: nucleic acid extraction | If any DNA quantification methods were used prior to DNA amplification or at the pooling step of library preparation, state so here. | Yes | Lines 103-104 |
| 4.5 | Human DNA sequence depletion or microbial DNA enrichment | Describe whether human DNA sequence depletion or enrichment of microbial or viral DNA was performed. | STORMS | | NA | |

| | | | | | | |
|---|---|---|---|---|---|---|
| 4.6 | Primer selection | Provide primer selection and DNA amplification methods as well as variable region sequenced (if applicable). | MIxS: pcr primers | | Yes | Lines 110-112 |
| 4.7 | Positive Controls | Describe any positive controls (mock communities) if used. | STORMS | If used, should be deposited under guidance provided in the 8.X items. | Yes | Line 114; supplemental methods |
| 4.8 | Negative Controls | Describe any negative controls if used. | STORMS | If used, should be deposited under guidance provided in the 8.X items. | Yes | Line 114; supplemental methods |
| 4.9 | Contaminant mitigation and identification | Provide any laboratory or computational methods used to control for or identify microbiome contamination from the environment, reagents, or laboratory. | STORMS | Includes filtering of reagents and other steps to minimize contamination. It is relevant to state whether the specimens of interest have low microbial load, which makes contamination especially relevant. | Yes | Supplemental methods |
| 4.10 | Replication | Describe any biological or technical replicates included in the sequencing, including which steps were replicated between them. | STORMS | Replication may be biological (redundant biological specimens) or technical (aliquots taken at different stages of analysis) and used in extraction, sequencing, preprocessing, and/or data analysis. | Yes | Supplemental methods |
| 4.11 | Sequencing strategy | Major divisions of strategy, such as shotgun or amplicon sequencing. | MIxS: sequencing method | For amplicon sequencing (for example, 16S variable region), state the region selected. State the model of sequencer used. | Yes | Supplemental methods |
| 4.12 | Sequencing methods | State whether experimental quantification was used (QMP/cell count based, spike-in based) or whether relative abundance methods were applied. | STORMS | These include read length, sequencing depth per sample (average and minimum), whether reads are paired, and other parameters. | Yes | Supplemental methods |
| 4.13 | Batch effects | Detail any blocking or randomization used in study design to avoid confounding of batches with exposures or outcomes. Discuss any likely sources of batch effects, if known. | STORMS | Sources of batch effects include sample collection, storage, library preparation, and sequencing and are commonly unavoidable in all but the smallest of studies. | Yes | Lines 164-167 |
| 4.14 | Metatranscriptomics | Detail whether any mRNA enrichment was performed and whether/how retrotranscription was performed prior to sequencing. Provide size range of isolated transcripts. Describe whether the sequencing library was stranded or not. Provide details on sequencing methods and platforms. | STORMS | Provide details on any internal standards which may have been used as well as parameters and versions of any software or databases used. | NA | |
| 4.15 | Metaproteomics | Detail which protease was used for digestion. Provide details on proteomic methods and platforms (e.g. LC-MS/MS, instrument type, column type, mass range, resolution, scan speed, maximum injection time, isolation window, normalised collision energy, and resolution). | STORMS | Provide details on any internal standards which may have been used as well as parameters and versions of any software or databases used. | NA | |
| 4.16 | Metabolomics | Specify the analytic method used (such as nuclear magnetic resonance spectroscopy or mass spectrometry). For mass spectrometry, detail which fractions were obtained (polar and/or non-polar) and how these were analyzed. Provide details on metabolomics methods and platforms (e.g. derivatization, instrument type, injection type, column type and instrument settings). | STORMS | Provide details on any internal standards which may have been used as well as parameters and versions of any software or databases used. | NA | |
| 5.0 | Data sources/ measurement | For each non-microbiome variable, including the health condition, intervention, or other variable of interest, state how it was defined, how it was measured or collected, and any transformations applied to the variable prior to analysis. | MIxS: host disease status | State any sources of potential bias in measurements, for example multiple interviewers or measurement instruments, and whether these potential biases were assessed or accounted for in study design.<br>Use terms from a standardized ontology such as the Experimental Factor Ontology (https://www.ebi.ac.uk/efo/) to describe variables of interest in a standardized format. | Yes | Lines 87-96; lines 122-132 |
| 6.0 | Research design for causal inference | Discuss any potential for confounding by variables that may influence both the outcome and exposure of interest. State any variables controlled for and the rationale for controlling for them. | STORMS | For causal inference, this item refers to describing the assumptions that would be required to draw causal inferences from observational data. See Vujkovic-Cvijin, I., Sklar, J., Jiang, L. et al. Host variables confound gut microbiota studies of human disease. Nature 587, 448–454 (2020). https://doi.org/10.1038/s41586-020-2881-9 for more details on confounding in observational microbiome studies.<br>For example, hypothesized confounders may be controlled for by multivariable adjustment. Consider using a directed acyclic graph (DAG) to describe your causal model and justify any variables controlled for. DAGs can be made using www.dagitty.net. | Yes | Lines 164-167; lines 211-213; lines 223-225; lines 486-488 |

| | | | | | | |
|---|---|---|---|---|---|---|
| 6.1 | Selection bias | Discuss potential for selection or survival bias. | STORMS | Selection bias can occur when some members of the target study population are more likely to be included in the study/final analytic sample than others. Some examples include survival bias (where part of the target study population is more likely to die before they can be studied), convenience sampling (where members of the target study population are not selected at random), and loss to follow-up (when probability of dropping out is related to one of the things being studied). | Yes | Lines 483-485 |
| 7.0 | Bioinformatic and Statistical Methods | Describe any transformations to quantitative variables used in analyses (e.g. use of percentages instead of counts, normalization, rarefaction, categorization). | STORMS | If a variable is analyzed using different transformations, state rationale for the transformation and for each analyses which version of the variable is used.<br>In case of any complex or multistep transformations, give enumerated instructions for reproducing those transformations. | Yes | Lines 129-130; 134-155 |
| 7.1 | Quality Control | Describe any methods to identify or filter low quality reads or samples. | MIxS: sequence quality check | If samples were excluded based on quality or read depth, list the criteria used, the number of samples excluded, and the final sample size after quality control. | Yes | Lines 115-117; supplemental methods |
| 7.2 | Sequence analysis | Describe any taxonomic, functional profiling, or other sequence analysis performed. | MIxS: feature prediction; similarity search method | | Yes | Lines 117-118; supplemental methods |
| 7.3 | Statistical methods | Describe all statistical methods. | Modified STROBE | Describe any statistical tests used, exploratory data analysis performed, dimension reduction methods/unsupervised analysis, alpha/beta metrics, and/or methods for adjusting for measurement bias.<br>If multiple statistical methods are possible, discuss why the methods used were selected.<br>If a multiple hypothesis testing correction method was used, describe the type of correction used.<br>State which taxonomic levels are analyzed. | Yes | Lines 134-229 |
| 7.4 | Longitudinal analysis | If the study is longitudinal, include a section that explicitly states what analysis methods were used (if any) to account for grouping of measurements by individual or patterns over time. | STORMS | | NA | |
| 7.5 | Subgroup analysis | Describe any methods used to examine subgroups and interactions. | STROBE | | NA | |
| 7.6 | Missing data | Explain how missing data were addressed. | STROBE | "Missing data" refers to participant measurements such as covariates, exposures, outcomes, or time points that should have been collected but were not, not to zeros in taxonomic abundance tables or data points not applicable to that observation. | Yes | Lines 156-157 |
| 7.7 | Sensitivity analyses | Describe any sensitivity analyses. | STROBE | | Yes | Lines 192-196 |
| 7.8 | Findings | State criteria used to select findings for reporting. | STORMS | For example, false discovery rate with total number of tests, effect size threshold, significance threshold, microbes of interest. | Yes | Lines 169-172; lines 181-183; lines 214-215; lines 226-228 |
| 7.9 | Software | Cite all software (including read mapping software) and databases (including any used for taxonomic reference or annotating amplicons, if applicable) used. Include version numbers. | Modified STREGA | Installed packages, add-ons or libraries should be stated and cited in addition to the software used.<br>All parameters employed that differ from the default of that software/version should be provided.<br>This is in addition to, not a replacement for, publishing of code as outlined in the section Reproducible Research. | Yes | Lines 129-132; 163-164; 178-179; 205-206; 218-220; 223-228; supplemental methods |

| | | | | | | |
|---|---|---|---|---|---|---|
| 8.0 | Reproducible research | Make a statement about whether and how others can reproduce the reported analysis. | STORMS | Any protected information that has been excluded or provided under controlled access should be listed along with any relevant data access procedures. "On request from authors" is not sufficiently detailed; formal data access procedures and conditions should be defined.<br>If data are unavailable, state so clearly.<br>Consider using a specialized rubric for reproducible research (such as: https://mbio.asm.org/content/9/3/e00525-18.short).<br>Consider preregistering the study protocol (such as on osf.io or https://plos.org/open-science/preregistration/). | Yes | Lines 541-551 |
| 8.1 | Raw data access | State where raw data may be accessed including demultiplexing information. | STORMS | Robust, long-term databases such as those hosted by NCBI and EBI are preferred. If using a private repository, provide rationale. | Yes | Lines 541-551 |
| 8.2 | Processed data access | State where processed data may be accessed. | STORMS | Unfiltered data should be provided.<br><br>Robust, long-term databases such as those hosted by NCBI and EBI-EMBL are preferred. Repositories like zenodo (https://zenodo.org/) or publisso (https://www.publisso.de/en/working-for-you/doi-service/) can be used to provide a DOI and long-term storage for processed datasets, even those which cannot be published openly. | Yes | Lines 541-551 |
| 8.3 | Participant data access | State where individual participant data such as demographics and other covariates may be accessed, and how they can be matched to the microbiome data. | STORMS | If re-categorized, transformed, or otherwise derived variables were used in the analysis, these variables or code for deriving them should be provided.<br>Examples of how participant data can be matched to microbiome data are: using the same set of anonymized identifiers, or using different anonymized identifiers but providing a map.<br>Provided data should be sufficient to independently replicate the current analysis. | Yes | Lines 541-551 |
| 8.4 | Source code access | State where code may be accessed. | STORMS | If a standard or formalized workflow was employed, reference it here. | Yes | Lines 541-551 |
| 8.5 | Full results | Provide full results of all analyses, in computer-readable format, in supplementary materials. | STORMS | For example, any fold-changes, p-values, or FDR values calculated, provided as a spreadsheet.<br>Use a machine-readable, plain-text format such as csv or tsv. | Yes | Supplemental Tables S1-S7 |
| **Results** | | | | | | |
| 9.0 | Descriptive data | Give characteristics of study participants (e.g. dietary, demographic, clinical, social) and information on exposures and potential confounders. | STROBE | Typically reported in a table included in the paper or as a supplementary table. Indicate number of participants with missing data for each variable of interest.<br>This includes environmental and lifestyle factors that may affect the relationship between the microbiome and the condition of interest. Participant diet and medication use should be summarized, if known.<br>At minimum, age and sex of all participants should be summarized. | Yes | Table 1 |
| 10.0 | Microbiome data | Report descriptive findings for microbiome analyses with all applicable outcomes and covariates. | STORMS | This includes measures of diversity as well as relative abundances. These descriptive findings should be reported both for the sample overall and for individual groups. | Yes | Table 1 |
| 10.1 | Taxonomy | Identify taxonomy using standardized taxon classifications that are sufficient to uniquely identify taxa. | STORMS | If not using full taxonomic hierarchy, make sure it is clear whether names stated are species, genera, family, etc.<br>Italicize genus/species pairs. Consult journal guidelines or standardized references on taxonomic nomenclature. For instance, https://wwwnc.cdc.gov/eid/page/scientific-nomenclature | Yes | Tables 2 and 3; Figures 2 and 6 |
| 10.2 | Differential abundance | Report results of differential abundance analysis by the variable of interest and (if applicable) by time, clearly indicating the direction of change and total number of taxa tested. | STORMS | If there are more than two groups, include omnibus (multigroup) test results if applicable to the research question.<br>If applicable, reported effect sizes should include a measure of uncertainty such as the confidence interval. | Yes | Lines 308-327 |
| 10.3 | Other data types | Report other data analyzed--e.g. metabolic function, functional potential, MAG assembly, and RNAseq. | STORMS | | NA | |
| 10.4 | Other statistical analysis | Report any statistical data analysis not covered above. | STORMS | This could include subgroup analysis, sensitivity analyses, and cluster analysis. | Yes | Lines 339-364 |

| | | | | | | |
|---|---|---|---|---|---|---|
| | | | | Visualizations should be easily interpretable and colorblind-friendly. The caption and/or main text should provide a detailed description of visualizations for visually-impaired readers. | | |
| **Discussion** | | | | | | |
| 11.0 | Key results | Summarise key results with reference to study objectives | STROBE | | Yes | Lines 367-379 |
| 12.0 | Interpretation | Give a cautious overall interpretation of results considering objectives, limitations, multiplicity of analyses, results from similar studies, and other relevant evidence. | STROBE | Define or clarify any subjective terms such as "dominant," "dysbiosis," and similar words used in interpretation of results.<br>When interpreting the findings, consider how the interpretation of the findings may be summarized or quoted for the general public such as in press releases or news articles.<br>If causal language is used in the interpretation (such as "alters," "affects," "results in," "causes," or "impacts"), assumptions made for causal inference should be explicitly stated as part of 6.0 and 13.0.<br>Distinguish between function potential (ie inferred from metagenomics) and observed activity (ie metatranscriptomic, metabolomic, proteomic) if discussing microbial function. | Yes | Lines 196-288 |
| 13.0 | Limitations | Discuss limitations of the study, taking into account sources of potential bias or imprecision. | STROBE | Also consider limitations resulting from the methods (especially novel methods), the study design, and the sample size. | Yes | Lines 477-502 |
| 13.1 | Bias | Discuss any potential for bias to influence study findings. | STORMS | May include sampling method, representativeness of study participants, or potential confounding. | Yes | Lines 483-484 |
| 13.2 | Generalizability | Discuss the generalisability (external validity) of the study results | STROBE | To what populations or other settings do you expect the conclusions to generalize? | Yes | Line 485-486 |
| 14.0 | Ongoing/future work | Describe potential future research or ongoing research based on the study's findings. | STORMS | | | Lines 516-518 |
| **Other information** | | | | | | |
| 15.0 | Funding | Give the source of funding and the role of the funders for the present study and, if applicable, for the original study on which the present article is based | STROBE | | Yes | Lines 555-556 |
| 15.1 | Acknowledgements | Include acknowledgements of those who contributed to the research but did not meet critera for authorship. | STORMS | For general guidelines on authorship, see http://www.icmje.org and https://www.elsevier.com/authors/journal-authors/policies-and-ethics/credit-author-statement | Yes | Lines 563-566 |
| 15.2 | Conflicts of Interest | Include a conflicts of interest statement. | STORMS | | Yes | Lines 553 |
| 16.0 | Supplements | Indicate where supplements may be accessed and what materials they contain. | STORMS | | Yes | Published with manuscript |
| 17.0 | Supplementary data | Provide supplementary data files of results with for all taxa and all outcome variables analyzed. Indicate the taxonomic level of all taxa. | STORMS | Depending on the analysis performed, examples of the supplemental results included could be mean relative abundance, differential abundance, raw p-value, multiple hypothesis testing-adjusted p-values, and standard error.<br>All discussed taxa should include the taxonomic level (e.g. class, order, genus). | | Supplemental Table S5 |

**Figure S1: Sample flowchart.**

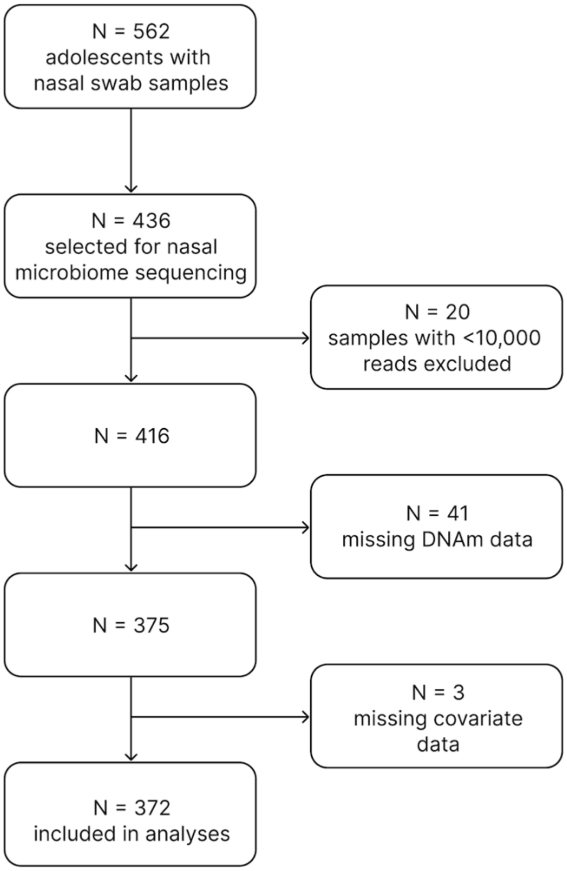

**Figure S2: Ordination plots of microbiome sample cluster analysis.** partitioning around medoids (PAM) was performed based on Jensen-Shannon divergence (JSD) and six clusters to maximize the clustering metrics.

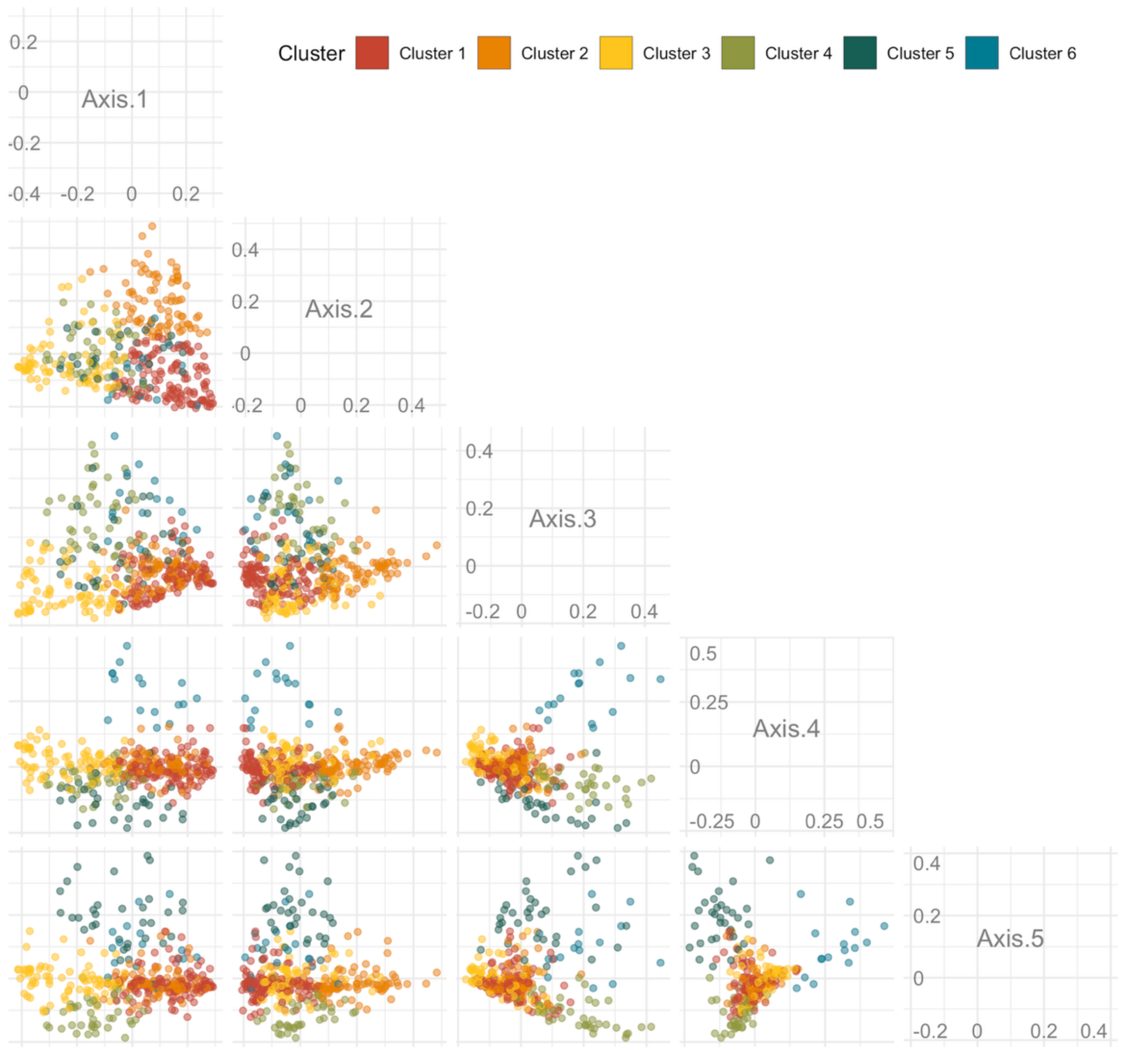

**Figure S3: Microbiome cluster assignments for leave-one-out testing.** Partitioning around medoids (PAM) was performed using leave-one-out for individual samples, and the leave-one-out sample was assigned to a cluster based on the medoid with the minimum distance.  The frequences of microbiome cluster assignments for each iteration are plotted.

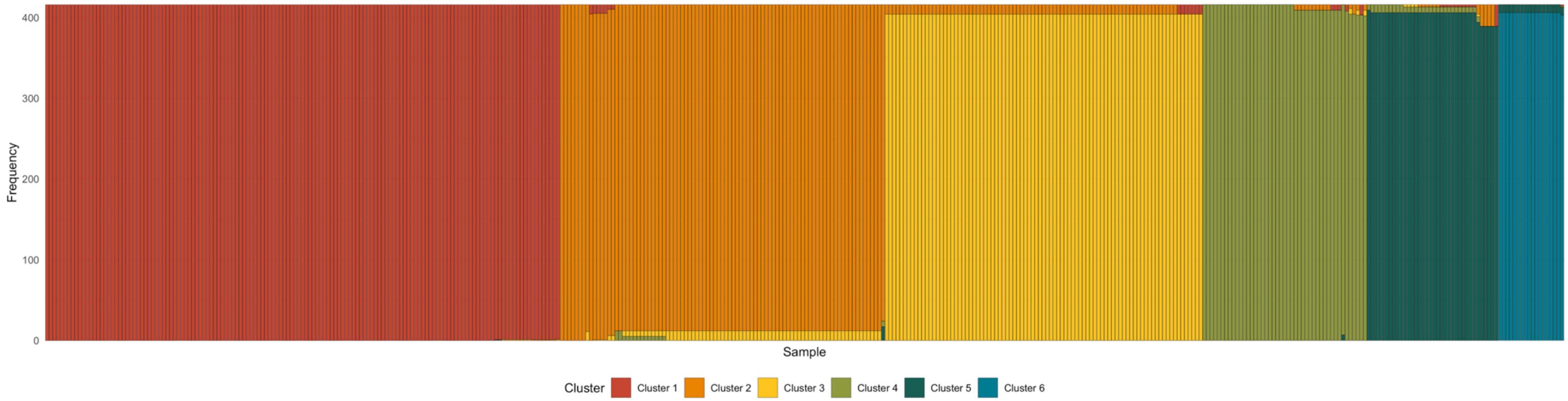

**Figure S4: Q-Q plots, volcano plots, and Manhattan plots.** Epigenome-wide association study of DNA methylation associated with microbiome cluster contrasts. Results from robust linear models adjusted for sine and cosine of season to control for annual and biannual trends, age at sample collection, race and ethnicity, BMI z-score, maternal education, 10 ReFACTor components, and 16S batch-run. Cluster 1: *Corynebacterium* dominant; Cluster 2: *Propionibacterium* dominant; Cluster 3: *Staphylococcus* dominant; Cluster 4: *Staphylococcus and Streptococcus* dominant; Cluster 5: unclassified *Neisseriaceae* dominant; and Cluster 6: *Moraxella* dominant.

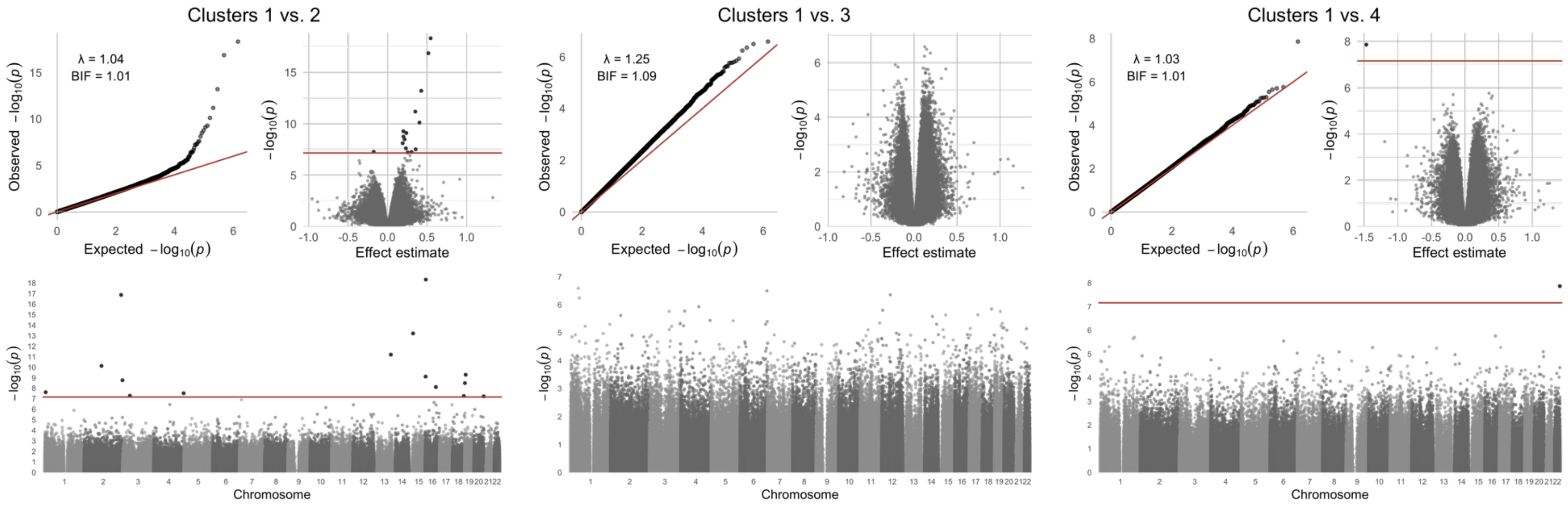

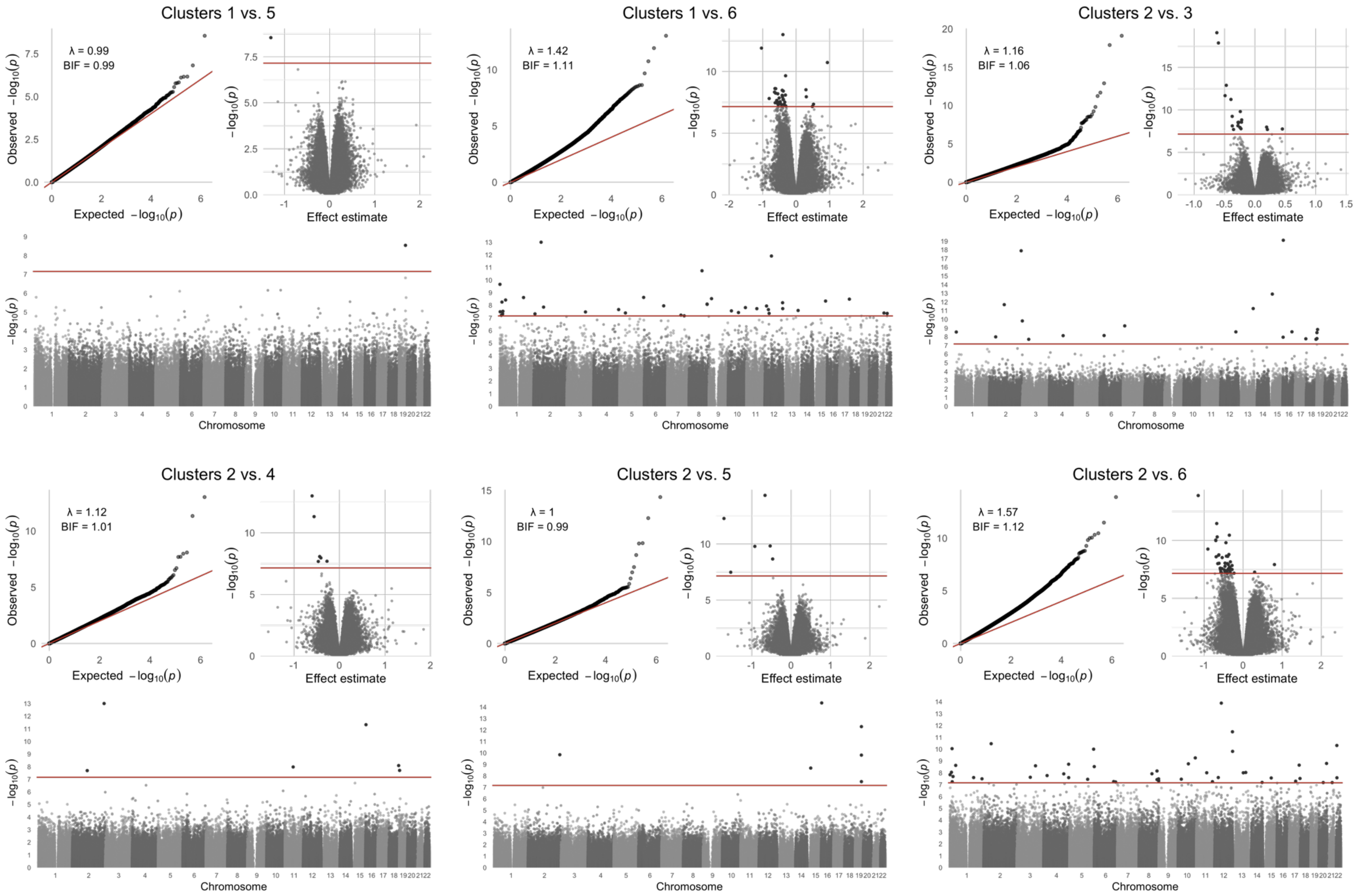

Clusters 1 vs. 5
λ = 0.99
BIF = 0.99
Observed −log10(p)
Expected −log10(p)
−log10(p)
Effect estimate
Chromosome
Clusters 1 vs. 6
λ = 1.42
BIF = 1.11
Clusters 2 vs. 3
λ = 1.16
BIF = 1.06
Clusters 2 vs. 4
λ = 1.12
BIF = 1.01
Clusters 2 vs. 5
λ = 1
BIF = 0.99
Clusters 2 vs. 6
λ = 1.57
BIF = 1.12

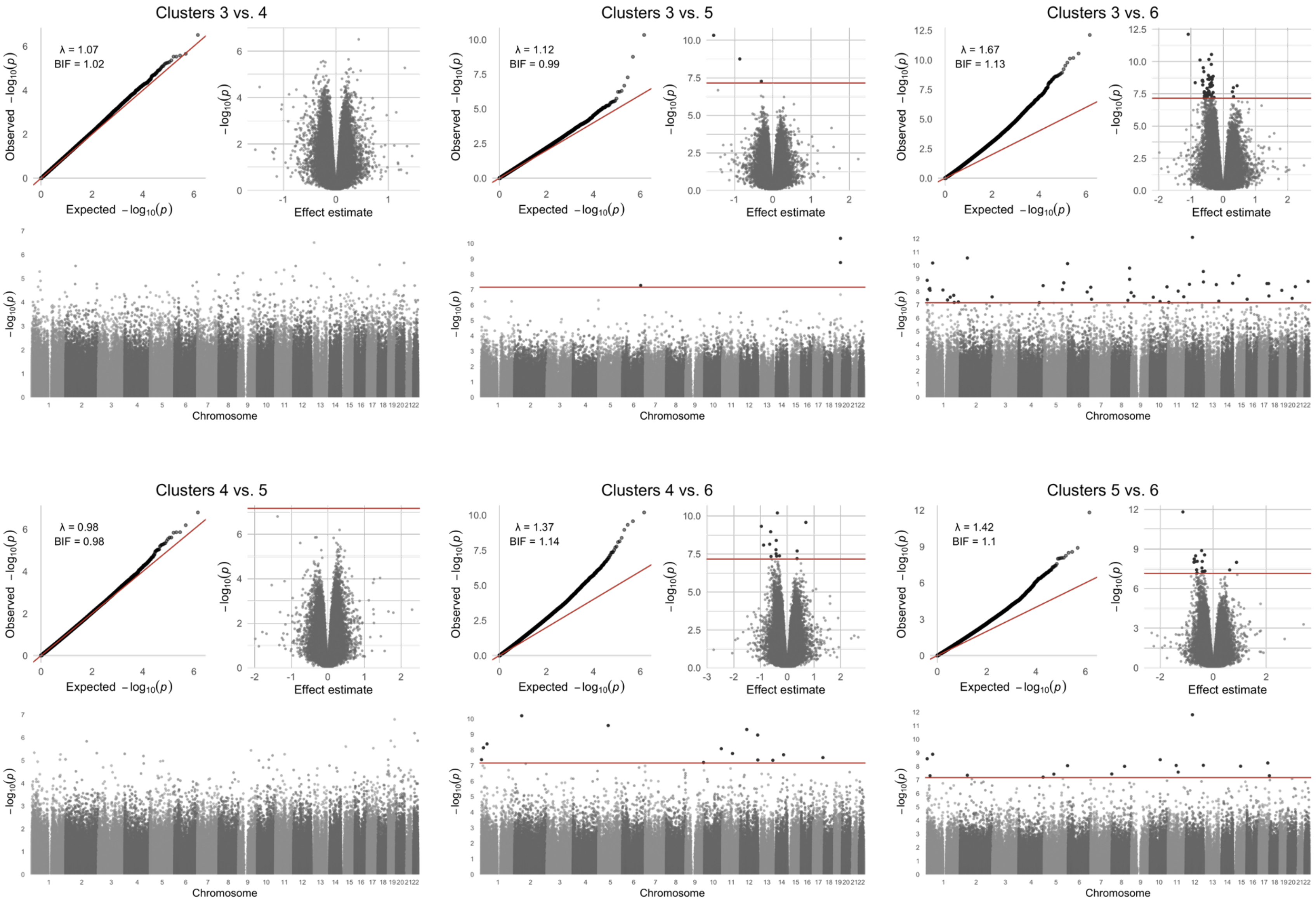

Clusters 3 vs. 4
λ = 1.07
BIF = 1.02
Clusters 3 vs. 5
λ = 1.12
BIF = 0.99
Clusters 3 vs. 6
λ = 1.67
BIF = 1.13
Clusters 4 vs. 5
λ = 0.98
BIF = 0.98
Clusters 4 vs. 6
λ = 1.37
BIF = 1.14
Clusters 5 vs. 6
λ = 1.42
BIF = 1.1
Observed −log10(p)
Expected −log10(p)
−log10(p)
Effect estimate
Chromosome

**Figure S5: Beta-values of differentially methylated positions (DMPs) ($p_{Bonferroni}$ < 0.05).** Cluster 1: *Corynebacterium* dominant; Cluster 2: *Propionibacterium* dominant; Cluster 3: *Staphylococcus* dominant; Cluster 4: *Staphylococcus and Streptococcus* dominant; Cluster 5: unclassified *Neisseriaceae* dominant; and Cluster 6: *Moraxella* dominant.

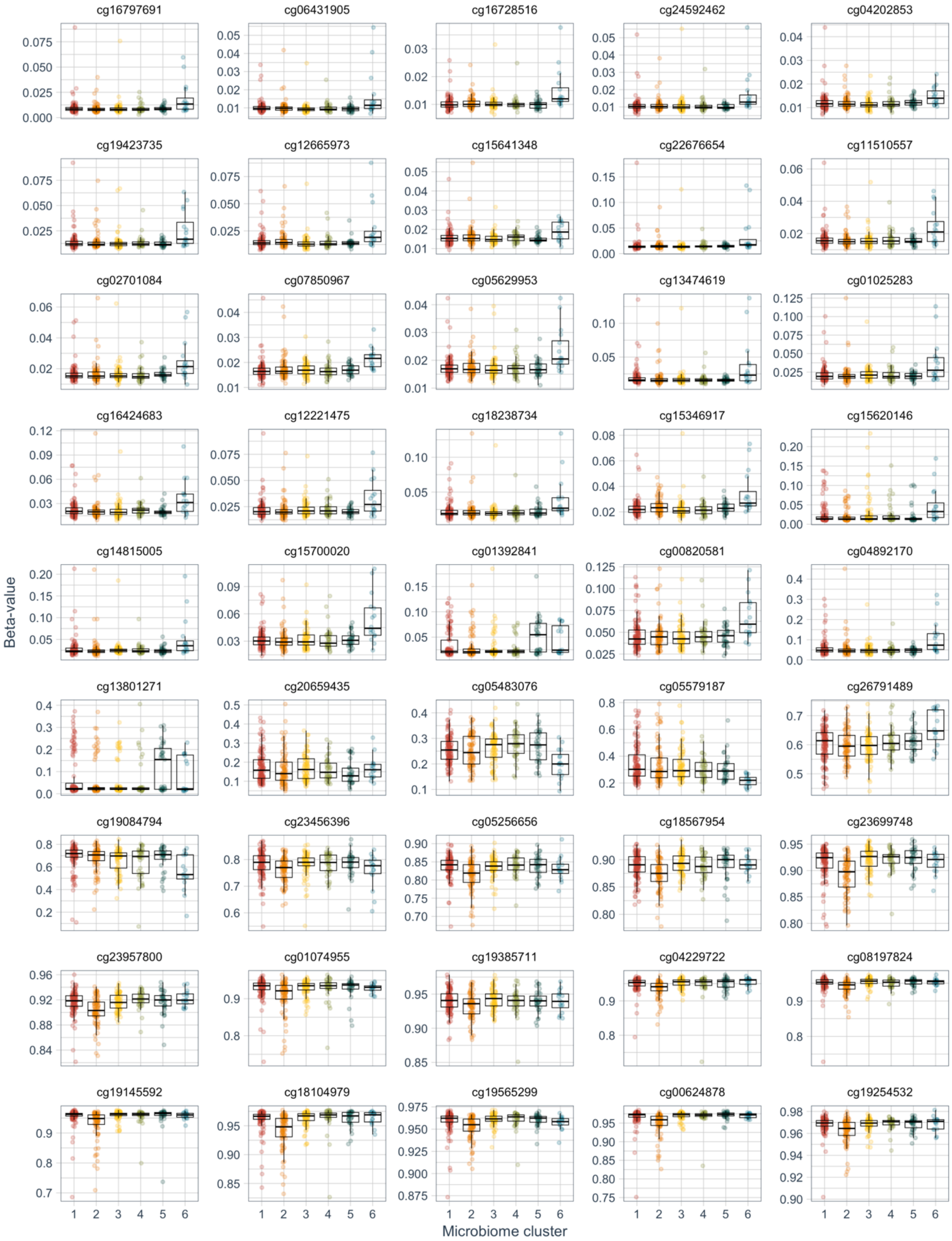

**Figure S6: Beta values of cg01392841, cg13801271, and cg15825916 (intergenic, chromosome 19).** Individual samples connected by gray lines.

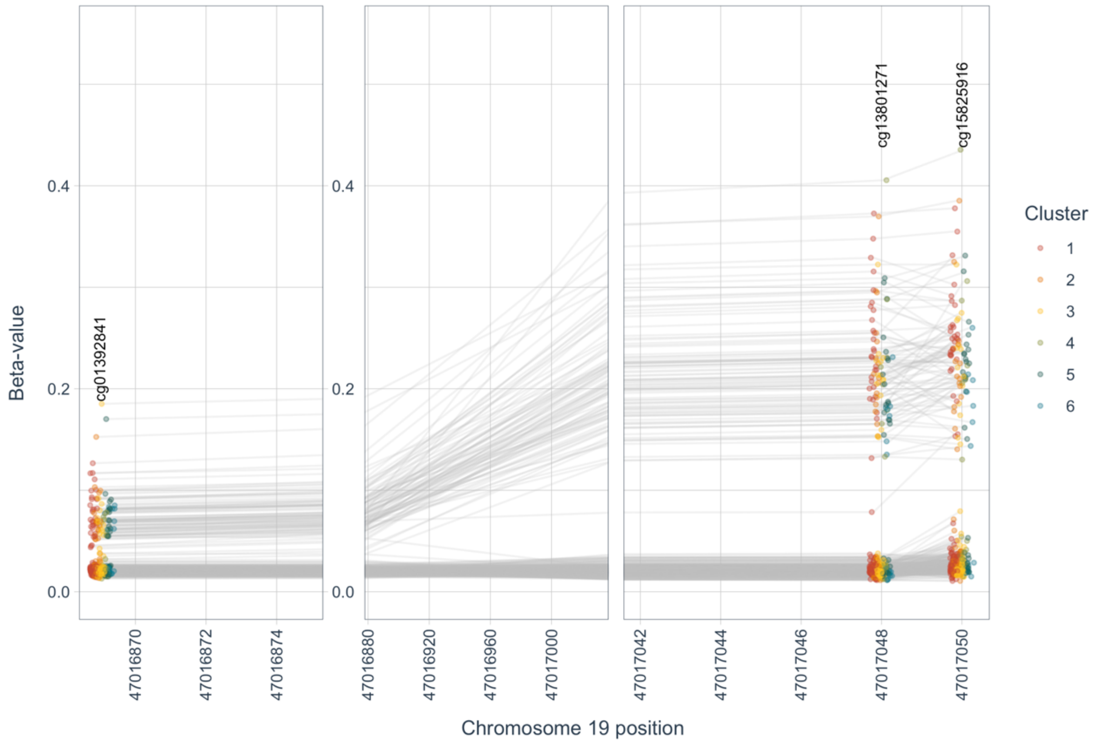

**Figure S7: Volcano plot for differential abundance analysis of Horvath epigenetic age deviation (EAD).** Results from analyses adjusted for sine and cosine of season, sex, age, race and ethnicity, BMI z-score, maternal education, and 16S batch-run. The association with *FDR* < 0.05 and that passed sensitivity analyses is shown as a labeled black point.

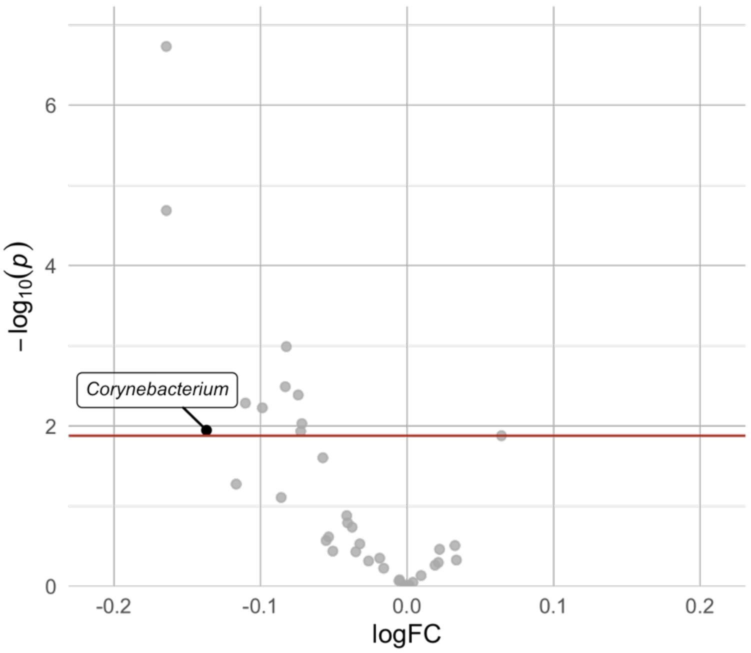

**Figure S8: Histogram of number of differentially methylated positions (DMPs) from permuted datasets ($p_{Bonferroni}$ < 0.05 for pairwise contrasts).** Results from 500 permutations of fully adjusted EWAS with random cluster assignments. The number of observed DMPs observed in the real dataset is shown as the red vertical line.

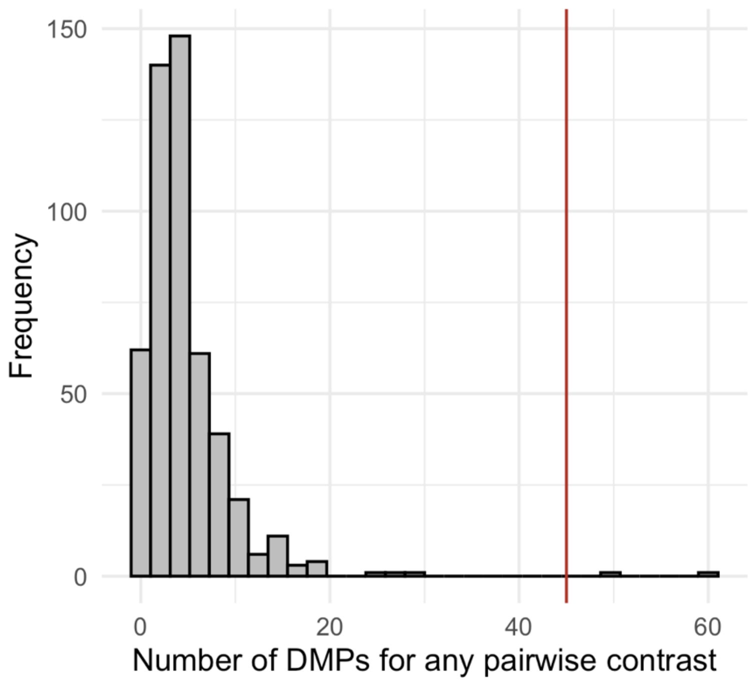

Supplement: Supplementary file 1 — Supplementary Material 1. [file 13148_2026_2093_MOESM1_ESM.pdf]
