# Supplementary material for: Interplay of the nasal microbiome and epigenome among adolescents
Source: Clin Epigenetics. 2026 Feb 27;18:93. doi: 10.1186/s13148-026-02093-1 (PMC13202811; doi:10.1186/s13148-026-02093-1)
Supplement: Supplementary file 3 — Supplementary Material 3. [file 13148_2026_2093_MOESM3_ESM.pdf]

Supplemental Material

Title: Interplay of the nasal microbiome and epigenome among adolescents

Authors: Anne K. Bozack, Javier Perez-Garcia, Sheryl Rifas-Shiman, Yanjiao Zhou, Joanne Sordillo, Jenny Jyoung Lee, Brent Coull, Peggy S. Lai, Emily Oken, Marie-France Hivert, Diane R. Gold, Andres Cardenas

Nasal microbiome sequencing

Microbiome sequencing was conducted at the Jackson Laboratory (Farmington, CT). Twenty-one samples were run twice due to low read counts, and the run with the higher read count was retained for analysis. The V1-V3 regions of bacterial 16S ribosomal RNA (rRNA) gene were amplified from genomic DNA using 27F (5'-AGAGTTTGATCCTGGCTCAG-3') and 534R primers (5'-ATTACCGCGGCTGCTGG-3'). 16S libraries were sequenced using an Illumina MiSeq and 2x300 v3 sequencing kit (Illumina, San Diego, CA) with dual distinct barcodes. Negative extraction and library controls were nuclease free water. Positive extraction and library controls were *Staphylococcus aureus* and *Escherichia coli*, respectively.

Initial processing of raw sequencing data was performed using Illumina's software. Sample demultiplexing was based on one mismatch in primer and zero mismatch in barcodes. Sequences with low quality (average < 35) and ambiguous bases (Ns) were removed. Data were further processed by removing 16S primers using the Trimomatic program [1] and paired-end sequences were assembled using the FLASH algorithm [2]. Chimeric sequences were removed using UChime [3] with "Gold" databases for 16S amplicons according to the UChime documentation. Using the USEARCH algorithm [4], 16S amplicons were clustered into operational taxonomic units (OTUs) at 97% sequence identity levels. The taxonomic classification was identified for each OTU representative sequence and sequence for every sample using the RDP Classifier [5]. OTU data were processed using the *phyloseq R* package [6]. OTUs classified as mitochondria or chloroplast and singletons were dropped. We performed decontamination using the *decontam R* package, which identifies contaminants based on the prevalence of OTUs in negative controls compared with positive samples, using a probability threshold of 0.1. We visually

assessed samples for outliers using PCoA plots. Based on rarefaction plots, we dropped samples with <10,000 reads (N = 20), leaving 416 high-quality samples available for analysis. We also dropped low abundance OTUs, defined as having <10 copies in individual samples [7] (0.57% of reads).

DNA methylation measurement

Extracted DNA samples were randomized to 96-well plates by sex and status of asthma and allergic rhinitis to minimize batch effects. Following bisulfite conversion, DNAm was measured using the Infinium MethylationEPICv1 BeadChip (Illumina, San Diego, CA, US), which interrogates >850,000 CpG sites. We preprocessed DNAm data using the *minfi* R package [8]. Sample-level quality control included dropping samples with low intensities (n = 3), samples with a mismatch between recorded and predicted sex (n = 4), and samples that failed a genotype check (n = 8). After exclusion of 35 technical replicates, DNAm data were available for 547 samples.

We calculated detection p-values based on control probe intensities and excluded probes with p-values > 0.05 for ≥ 5% of samples. We performed functional normalization using three principal components (PCs) from control probes to remove technical variability [9] and regression on correlated probes to adjust for probe-type bias [10]. To adjust for batch effects due to sample plate, we used *ComBat* from the *sva* R package [11]. We excluded probes annotated to sex chromosomes, non-CpG probes, probes with a SNP with minor allele frequency (MAF) > 5% within two base pairs of the target site or single base pair extension based on the *rmSNPandCH* function from *DMRcate* [12], probes with a SNP at the target site or single base pair extension with a MAF > 5% in admixture American or European populations based on McCartney et al. [13], and cross-reactive probes based on McCartney et al. and Pidsley et al. [14]. We additionally identified probes targeting SNPs based on the distribution of Beta-values using the *MethylToSNP* package [15]. Following probe-level quality control, 715,023 CpG sites were available for analysis. We visually inspected data using density distributions and PC plots to evaluate differences by technical and biological variables using *ENmix* [16]. To control for variation in cell type composition between samples, we used Reference-Free Adjustment for Cell-Type composition (ReFACTor), which is based on sparse PCA [17].

References

1. Bolger AM, Lohse M, Usadel B. Trimmomatic: a flexible trimmer for Illumina sequence data. Bioinformatics. 2014;30:2114–20. https://doi.org/10.1093/bioinformatics/btu170

2. Magoč T, Salzberg SL. FLASH: fast length adjustment of short reads to improve genome assemblies. Bioinformatics. 2011;27:2957–63. https://doi.org/10.1093/bioinformatics/btr507

3. Edgar RC, Haas BJ, Clemente JC, Quince C, Knight R. UCHIME improves sensitivity and speed of chimera detection. Bioinformatics. 2011;27:2194–200. https://doi.org/10.1093/bioinformatics/btr381

4. Edgar RC. Search and clustering orders of magnitude faster than BLAST. Bioinformatics. 2010;26:2460–1. https://doi.org/10.1093/bioinformatics/btq461

5. Wang Q, Garrity GM, Tiedje JM, Cole JR. Naive Bayesian classifier for rapid assignment of rRNA sequences into the new bacterial taxonomy. Appl Environ Microbiol. 2007;73:5261–7. https://doi.org/10.1128/AEM.00062-07

6. McMurdie PJ, Holmes S. phyloseq: an R package for reproducible interactive analysis and graphics of microbiome census data. PLoS One. United States; 2013;8:e61217. https://doi.org/10.1371/journal.pone.0061217

7. Nikodemova M, Holzhausen EA, Deblois CL, Barnet JH, Peppard PE, Suen G, et al. The effect of low-abundance OTU filtering methods on the reliability and variability of microbial composition assessed by 16S rRNA amplicon sequencing. Front Cell Infect Microbiol. 2023;13:1165295. https://doi.org/10.3389/fcimb.2023.1165295

8. Aryee M, Jaffe A, Corrada-Bravo H, Ladd-Acosta C, Feinberg A, Hansen K, et al. Minfi: a flexible and comprehensive Bioconductor package for the analysis of Infinium DNA methylation microarrays. Bioinformatics. 2014;30:1363–9. https://doi.org/10.1093/bioinformatics/btu049

9. Fortin J-P, Labbe A, Lemire M, Zanke BW, Hudson TJ, Fertig EJ, et al. Functional normalization of 450k methylation array data improves replication in large cancer studies. Genome Biol. 2014;15:503. https://doi.org/10.1186/s13059-014-0503-2

10. Xu Z, Langie SAS, De Boever P, Taylor JA, Niu L. RELIC: A novel dye-bias correction method for Illumina Methylation BeadChip. BMC Genomics. BioMed Central Ltd.; 2017;18:4. https://doi.org/10.1186/s12864-016-3426-3

11. Leek JT, Johnson WE, Parker HS, Jaffe AE, Storey JD. The sva package for removing batch effects and other unwanted variation in high-throughput experiments. Bioinformatics. 2012;28:882–3. https://doi.org/10.1093/bioinformatics/bts034

12. Peters TJ, Buckley MJ, Statham AL, Pidsley R, Samaras K, Lord RV, et al. De novo identification of differentially methylated regions in the human genome. Epigenetics Chromatin. 2015;8:6. https://doi.org/10.1186/1756-8935-8-6

13. McCartney DL, Walker RM, Morris SW, McIntosh AM, Porteous DJ, Evans KL. Identification of polymorphic and off-target probe binding sites on the Illumina Infinium MethylationEPIC BeadChip. Genomics Data. Elsevier; 2016;9:22–4. https://doi.org/10.1016/j.gdata.2016.05.012

14. Pidsley R, Zotenko E, Peters TJ, Lawrence MG, Risbridger GP, Molloy P, et al. Critical evaluation of the Illumina MethylationEPIC BeadChip microarray for whole-genome DNA methylation profiling. Genome Biol. BioMed Central; 2016;17:208. https://doi.org/10.1186/s13059-016-1066-1

15. LaBarre BA, Goncearenco A, Petrykowska HM, Jaratlerdsiri W, Bornman MSR, Hayes VM, et al. MethylToSNP: identifying SNPs in Illumina DNA methylation array data. Epigenetics Chromatin. 2019;12:79. https://doi.org/10.1186/s13072-019-0321-6

16. Xu Z, Niu L, Li L, Taylor JA. ENmix: a novel background correction method for Illumina HumanMethylation450 BeadChip. Nucleic Acids Res. 2016;44:e20. https://doi.org/10.1093/nar/gkv907

17. Rahmani E, Zaitlen N, Baran Y, Eng C, Hu D, Galanter J, et al. Sparse PCA corrects for cell type heterogeneity in epigenome-wide association studies. Nat Methods. 2016;13:443–5. https://doi.org/10.1038/nmeth.3809
